# FGFR1 but not S6K1/2 drives intrinsic BRAF inhibitor resistance in melanoma

**DOI:** 10.1038/s41420-026-03155-2

**Published:** 2026-05-19

**Authors:** Marwan Almoiliqy, Peng Li, Young-Ho Jung, Dorothy Zheng, Abeer Alshambky, Mohamad K. I. Koleilat, Michael A. Davies, Lawrence N. Kwong

**Affiliations:** 1 Department of Translational Molecular Pathology, The University of Texas MD Anderson Cancer Center, Houston, TX 77030 USA; 2 Department of Melanoma Medical Oncology, The University of Texas MD Anderson Cancer Center, Houston, TX 77030 USA; 3 Department of Genomic Medicine, The University of Texas MD Anderson Cancer Center, Houston, TX 77030 USA

**Keywords:** Melanoma, Targeted therapies

## Abstract

A decrease in phosphorylation of S6 has been identified by many studies as one of the top markers of response to several kinase inhibitors in cancer, especially BRAF inhibitors (BRAFi) in melanoma. However, it is unknown whether it is merely a marker or whether it plays a functional role in resistance. We therefore sought to comprehensively assess the role of the primary S6 kinases S6K1 and S6K2 in intrinsic BRAFi resistance, an understudied topic compared to acquired BRAFi resistance. Surprisingly, we found that overexpression or double knockout of S6K1/2 had no effect on BRAFi sensitivity or resistance in multiple melanoma cell lines. Double S6K1/2 knockout left a small amount of residual S6 phosphorylation, which we determined was at least partially maintained by CK1a. To determine whether pS6 itself affects BRAFi resistance, we overexpressed a phosphomimetic S6 construct in sensitive cell lines, but this also had no impact on BRAFi sensitivity. Instead, a pooled CRISPR kinome knockout screen uncovered FGFR1 as a key resistance driver, and indeed intrinsic BRAFi resistance was partially to fully reversible in 6 cell lines by the FDA-approved pan-FGFR inhibitor pemigatinib. Overall, our results suggest that although changes in pS6 are an excellent marker of intrinsic BRAFi resistance, the S6K1/2-S6 axis itself is not primarily responsible, and instead that FGFR1 is worth further translational study in the context of intrinsic BRAFi resistance.

## Introduction

Changes in S6 phosphorylation have been identified as a top biomarker of clinical and preclinical responses to BRAF and MEK inhibitors in melanoma [1], which are the standard of care for the ~50% of melanoma patients harboring a BRAF mutation. Specifically, a decrease in pS6 is strongly associated with sensitivity, while no change or an increase is associated with resistance. Such biomarkers are critical, given that 20–25% of BRAF-mutant melanoma patients are intrinsically resistant to BRAFi+MEKi [2] while 80–90% of initial responders eventually develop acquired resistance [3]. However, it is unknown whether S6 phosphorylation – and by extension, its direct kinases – are functionally involved in BRAFi resistance, or if it acts only as a marker.

Indeed, there is a preponderance of data identifying changes in pS6 as one of the top – if not the top – marker of response to MAPKi. S6 is phosphorylated at 5 functional serines: S235, S236, S240, S244, and S247 [4], though only the first four have so far been linked to drug sensitivity. In melanoma, changes in pS6 but not pERK nearly-perfectly segregated with response: 10/11 BRAFi-sensitive lines decreased pS6 upon BRAFi, while 5/5 intrinsically BRAFi-resistant lines had no changes in pS6 [1]. This extended to patient samples as well where decreases in pS6 significantly distinguished responders from non-responders. In two independent colon cancer studies, nearly identical in vitro results were found to melanoma: together, 12/12 MEKi-sensitive cell lines decreased pS6 upon MEKi, while 16/16 intrinsically resistant lines had no changes in pS6, whereas changes in pERK had no correlation to response [5, 6]. Similar results were found in HER2i-treated breast cancer cell lines [7]. Moreover, another study found that in 6 pairs of sensitive and acquired resistant cell lines, all 6 sensitive parental lines reduced pS6 upon BRAFi, but all 6 of their acquired resistant counterparts had no change in pS6 upon BRAFi [8]. Finally, pS6 is a marker of sensitivity to additional kinase inhibitors that target upstream of MAPK and PI3K including EGFRi, FGFRi, and SHP2i in lung cancer [9]. In total, these data show a strong correlation between S6 phosphorylation changes and MAPK inhibitor resistance.

BRAFi resistance (including BRAFi+MEKi) in particular can be divided into two types in melanoma: acquired, which arises de novo in response to therapy, and intrinsic, meaning the melanoma is insensitive to BRAFi upfront despite harboring a targetable BRAF mutation. Acquired resistance in patient melanoma samples is well-studied and is primarily due to the reactivation of the MAPK pathway through i) mutations in NRAS, KRAS, MEK1, or MEK2; ii) gain of BRAFi-resistant BRAF splice isoforms; or iii) amplification of BRAF [10–12]. The remaining types of acquired resistance either activate the PI3K pathway (PTEN, PIK3CA, or AKT1/3 mutations) or act through less well-characterized non-genomic mechanisms. Both MAPK and PI3K activate pS6. Intrinsic resistance, on the other hand, is far less well studied clinically; preclinically, however, intrinsically resistant cell lines are dominated by an AXL-HI/MITF-LO, EMT-like phenotype [13, 14] frequently accompanied by loss of PTEN. Indeed, AXL-targeted antibody-drug conjugates have shown some clinical promise [15], suggesting the phenotype is also present in patients. However, few preclinical studies make distinctions between intrinsic and acquired resistance.

The primary S6 kinases are the paralogs S6K1 and S6K2. We previously demonstrated that the S6K1-selective inhibitor PF-4708671 was sufficient to reverse acquired MAPKi resistance in vitro and in vivo in a model of melanoma [16]. Other studies have also implicated S6K1/2 in mediating kinase inhibitor sensitivity in other cancers: for example, S6K1 knockdown or pharmacological inhibition significantly enhanced EGFRi in lung cancer models in one study [17] and IGFRi in colon cancer models in another study [18], both in vitro and in vivo. Moreover, mTOR inhibition, which targets a step higher in the signaling cascade, decreases pS6 and re-sensitizes melanoma cell lines that have acquired BRAFi resistance [8]. Given these findings, plus the strong ability of changes in pS6 to separate intrinsically resistant and sensitive cell lines [1] and the paucity of treatments aimed at reversing intrinsic BRAFi resistance, we sought to more thoroughly interrogate pS6, S6K1, and S6K2 in this understudied setting.

Here, through a comprehensive series of genetic engineering and functional assays, we present data that show that S6K1 and S6K2 appear to be completely dispensable for intrinsic BRAFi resistance, and that neither S6K1/2 nor pS6 itself appear to be sufficient to induce BRAFi resistance, suggesting that pS6 acts primarily as a marker. Instead, a 763-gene CRISPR kinome screen identified FGFR1 as a driver of intrinsic BRAFi resistance, which could be reversed by the FDA-approved inhibitor pemigatinib in 6 melanoma cell lines. These results refine our understanding of intrinsic BRAFi resistance and present a new option for translational research.

## Results

### Selection of representative BRAFi sensitive and intrinsically resistant melanoma cell lines

We selected 6 representative cell lines to dissect the roles of S6K1 and S6K2: 3 sensitive lines, UACC257, MEL1617, and WM983b and 3 intrinsically resistant lines, 1205Lu, 2686, and RPMI-7951. All cell lines had a BRAF-V600E mutation and were wild type for NRAS, KRAS, and NF1. All three intrinsic resistant lines had a PTEN alteration of variable impact (Supplementary Table 1).

**Fig. 1:**
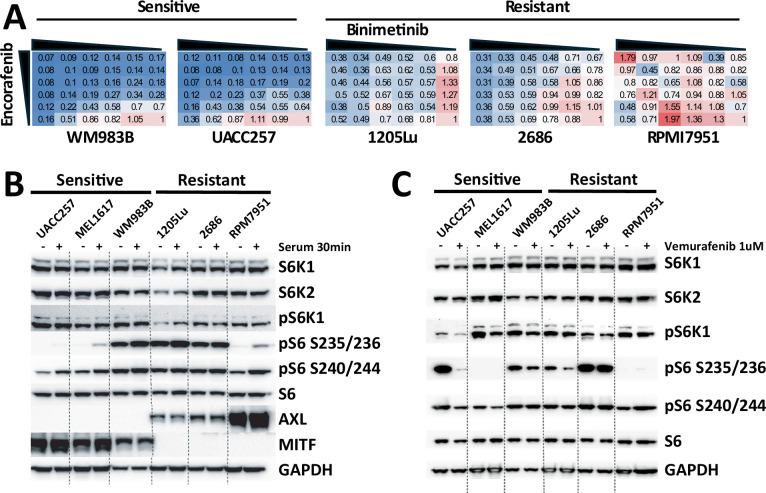
Melanoma cell lines selected for analysis. **A** Drug dose response matrix for BRAFi (encorafenib) and MEKi (binimetinib) for 5 of the cell lines. Doses are 0 nM, 10 nM, 30 nM, 100 nM, 300 nM, 1 μM. Numbers reflect the normalized OD590 values for crystal violet-stained cells. **B** Western blot of 6 cell lines at baseline: 3 sensitive and 3 intrinsically resistant to BRAFi. **C** Western blot of the same 6 cell lines in response to the BRAFi vemurafenib.

First, we confirmed the BRAFi sensitivity status of the lines using drug dose assays for the combination of BRAFi (encorafenib) and MEKi (binimetinib) (Fig. 1A). Next, consistent with known correlations [13, 14], we showed that the resistant lines expressed high AXL and low MITF, while the sensitive lines expressed low AXL and high MITF (Fig. 1B). Also consistent with previous reports, the baseline levels of S6K1, pS6K1, S6K2, S6, or pS6 did not correlate with sensitive or resistant status.

Next, we assessed the change in S6 phosphorylation in each line upon treatment with the BRAFi vemurafenib. Consistent with previous findings, all 3/3 sensitive lines showed a decrease in pS6-S235/236 and/or pS6-S240/244 in response to BRAFi (Fig. 1C). Conversely, 2/3 of the intrinsically resistant lines (2686 and RPMI-7951) showed no decrease in pS6 upon BRAFi, with 1205Lu showing a decrease only in pS6-S235/236. Thus, these cell lines largely recapitulate the known overall segregation of pS6 changes with BRAFi sensitivity.

### S6K1/2 overexpression does not confer BRAFi resistance

**Fig. 2:**
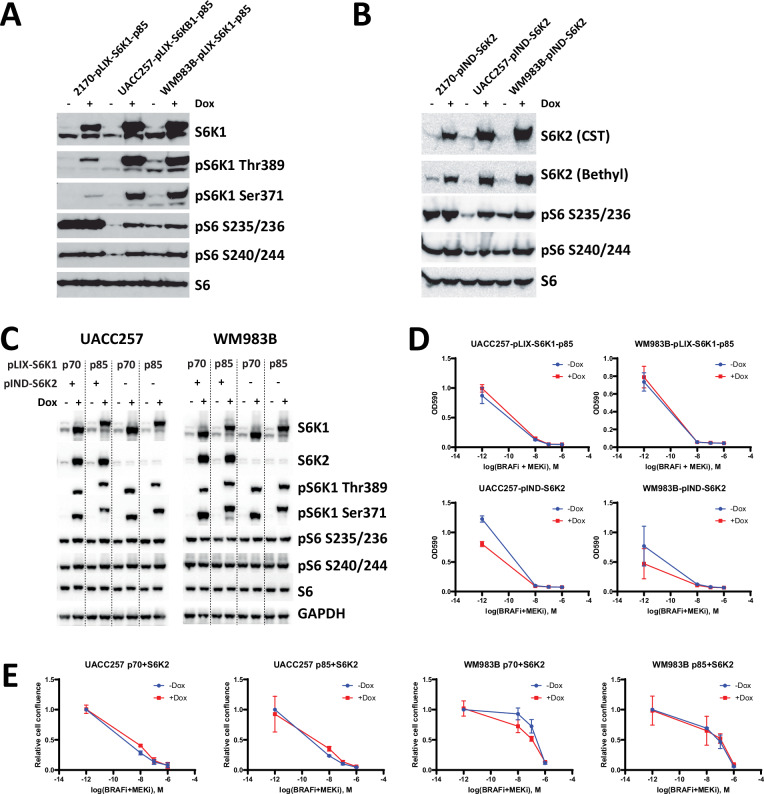
S6K1 and/or S6K2 overexpression modestly affects pS6 and has no effect on BRAFi sensitivity in BRAFi-sensitive cell lines. **A** Western blot for S6K1 and pS6 in the cell lines 2170, UACC257, and WM983B inducibly overexpressing p85 S6K1. **B** Western blot for S6K2 and pS6 in 2170, UACC257, and WM983B inducibly overexpressing S6K2. **C** Western blot for S6K1, S6K2, and pS6 in UACC257 and WM983B inducibly overexpressing both S6K1 (p70 or p85) and S6K2. **D** BRAFi+MEKi (encorafenib+binimetinib) dose response assay of UACC257 and WM983B inducibly overexpressing either S6K1 and S6K2. None of the doses showed statistical significance. **E** BRAFi+MEKi dose response assay of UACC257 and WM983B inducibly overexpressing both S6K1 (p70 or p85) and S6K2. The y-axis represents Incucyte Zoom automated confluence quantification, normalized to DMSO off dox. None of the doses showed statistical significance.

We first asked whether S6K1- or S6K2-induced phosphorylation of S6 is sufficient to confer BRAFi resistance. We overexpressed full-length p85 S6K1 or S6K2 in the BRAFi-sensitive cell lines UACC257 or WM983B, or the resistant line 2170. High overexpression of either kinase resulted in, at most, moderate increases in S6 phosphorylation in all 3 cell lines (Fig. 2A, B). We noted that the overexpressed S6K1 was actively phosphorylated (Fig. 2A); specific pS6K2 antibodies were unavailable.

We then overexpressed both S6K1, in either its p70 or p85 isoforms, and S6K2 together, using different antibiotic selection cassettes to ensure expression of both genes in all cells. Despite double overexpression, moderate to minimal increases in pS6 were observed at either S235/236 or S240/245 in either UACC257 or WM983B (Fig. 2C).

We then treated the cells with BRAFi and found that single or double S6K1/2 overexpression had no effect on BRAFi sensitivity in either UACC257 or WM983B, appearing indistinguishable from the drug responses in the absence of dox-induced gene expression (Fig. 2D, E). These data show that S6K1/2 overexpression is not sufficient to confer BRAFi resistance.

### S6K1/2 double knockout does not reverse intrinsic BRAFi resistance and leaves residual S6 phosphorylation

**Fig. 3:**
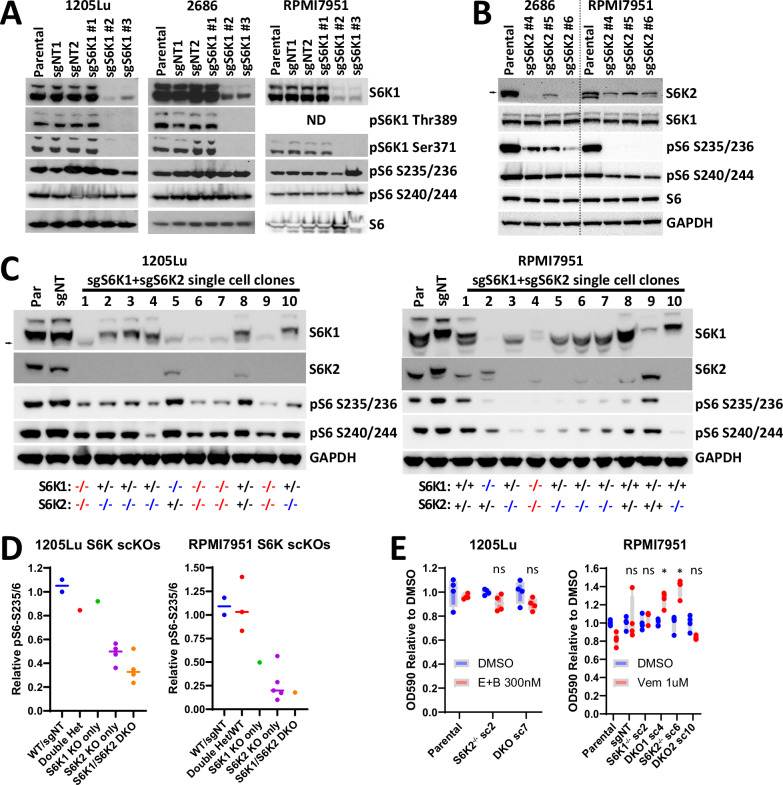
S6K1 and/or S6K2 knockout modestly affects pS6 and has no effect on BRAFi resistance in intrinsically BRAFi-resistant cell lines. **A** Western blot for S6K1 and pS6 in the BRAFi-resistant cell lines 1205Lu, 2686, and RPMI7951 with 3 different S6K1 sgRNAs. **B** Western blot for S6K2 and pS6 in 2686 and RPMI7951 with 3 different S6K2 sgRNAs. Arrow indicates non-specific band. **C** Western blots of 1205Lu or RPMI7951 cells transduced with both S6K1 and S6K2 sgRNAs, with each lane an independent single cell-cloned subline. Arrow indicates non-specific band. **D** Quantification of the blots in (**C**) for pS6-S235/6. **E** BRAFi assay of 1205Lu and RPMI7951 S6K1/2 single cell clones, as OD590 values of crystal violet staining after 4 days of treatment with DMSO or either 300 nM Encorafenib +300 nM Binimetinib for 1205Lu or 1 μM Vemurafenib for RPMI7951. For RPMI7951, sc2, sc4, and sc6 are the same as the ones in panel C, but DKO2 sc10 is from a separate set of single cell clones. ns, not significantly different compared to Parental, Student’s *t* test. *p < 0.05.

We next asked whether S6K1/2 might be necessary for intrinsic BRAFi resistance. We first generated polyclonal S6K1 knockouts in the intrinsically resistant cell lines 1205Lu, 2686, and RPMI-7951 by transduction with individual lentiviral CRISPR sgRNAs. Potent S6K1 KO by two independent successful sgRNAs had little to no effect on S6 phosphorylation in all 3 lines (Fig. 3A). We then knocked out S6K2 in 2686 and RPMI-7951. In contrast to S6K1, S6K2 KO by three independent successful sgRNAs generated profound decreases in pS6-S235/6 and weaker decreases in pS6-S240/244 (Fig. 3B).

We then sought to simultaneously achieve double S6K1/2 KO (DKO) and to also generate single cell-cloned KOs to obtain 100% homozygous loss of either or both genes. To do so, we transduced cells with both sgS6K1 and sgS6K2 simultaneously using different antibiotic selection cassettes. After single-cell subcloning, each subline uniformly harbored various combinations of wild type, heterozygous loss, or homozygous loss of S6K1 and/or S6K2. Complete protein loss in subclones with homozygous KO of either gene was confirmed by western blot in both 1205Lu and RPMI-7951 (Fig. 3C).

Direct comparison of the single S6K1 or S6K2 KOs confirmed that, consistent with the polyclonal KO data above, S6K1 loss by itself had little to moderate effects on pS6 levels, whereas S6K2 KO resulted in a strong decrease at S235/236 and a relatively weaker decrease at S240/244. Moreover, loss of S6K1 on top of S6K2 loss (i.e. DKOs) did not significantly further decrease pS6 levels compared to the S6K2 single KOs (Fig. 3D). Together, these data suggest that S6K2 is the primary S6 kinase in BRAF-mutant melanoma.

Finally, we tested the effect of S6K1/2 loss on BRAFi sensitivity and found no increase in the response to BRAFi in the single cell knockout clones versus parental or sgNT in all three cell lines 1205Lu, RPMI7951, and 2686 (Fig. 3E and Supplementary Fig. 1), indicating that complete loss of single S6K1 or S6K2, or of combined S6K1 and S6K2 are insufficient to reverse intrinsic BRAFi resistance in melanoma, and in two sublines may have even potentiated resistance.

### CK1a partially sustains S6 phosphorylation in the absence of S6K1/2

**Fig. 4:**
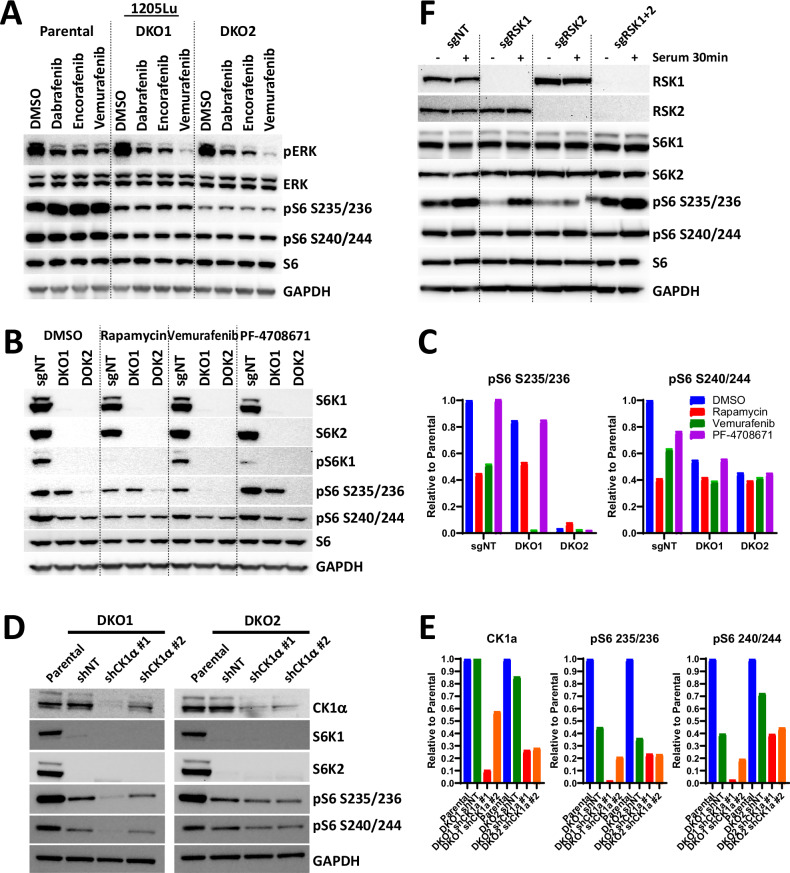
CK1a partially mediates residual S6 phosphorylation in S6K1/2 DKO cells. **A** Western blot of 1205Lu parental or DKO cells after 24 h of treatment with 3 different BRAFi, each at 1 μM. DKO1 and DKO2 are generated from different sgRNA combinations: DKO1 = S6K1 sgRNA #2 + S6K2 sgRNA#4; DKO2 = S6K1 sgRNA #3 + S6K2 sgRNA#6. **B** Western blot of 1205Lu parental or DKO cells after 24 h of treatment with mTORi (rapamycin), BRAFi (vemurafenib), or S6Ki (PF-4708671), each at 1 μM. **C** Quantification of the western blot in (**B**). As several pS6 S235/236 bands are close to the limit of detection by western blot, the relative differences should be interpreted with caution. **D** Western blot of 1205Lu parental, RSK1 KO, RSK2 KO, or RSK1/2 double KO cells with or without 30 min of serum treatment after 16 h of serum starvation. **E** Quantification of the western blot in (**D**). **F** Western blot of 1205Lu parental or DKO cells, with DKO cells receiving shNT or two different shCK1a constructs.

Despite a profound loss of pS6 in S6K1/2 DKO cells, we always detected at least a residual amount of pS6 in all cell lines, especially at S240/244. We therefore asked which kinases are responsible for this residual S6 phosphorylation. First, we determined that 3 different BRAFi did not further decrease pS6 in the DKO cells (Fig. 4A), indicating that the S6 kinase is not downstream of BRAF. We next tested rapamycin, which targets the mTOR complex upstream of multiple kinases [19] including S6K1/2 and RSKs [20], and PF-4708671, an S6K1/2 inhibitor. Similar to BRAFi, neither rapamycin nor PF-4708671 further decreased the residual pS6 S240/244 in DKOs and at most caused only a slight further decrease at S235/236 (Fig. 4B, C), suggesting that the responsible kinase(s) are largely independent of mTOR signaling.

CK1a (casein kinase 1 alpha) has previously been shown to phosphorylate S6 in HEK293T cells [21] and is not known as a downstream mTOR effector, making it an attractive candidate. To determine its role in residual S6 phosphorylation, we knocked down CK1a using shRNAs in our S6K1/2 DKO single-cell-cloned sublines. Approximately 50–70% knockdown of CK1a in DKO cells resulted in a further ~35% decrease in the residual pS6 S235/236 levels and a 40–45% decrease in pS6 S240/244, whereas a ~90% knockdown of CK1a resulted in a >90% loss of pS6, suggesting a dose dependency (Fig. 4D, E). Therefore, CK1a is at least partially responsible for the residual S6 phosphorylation observed in the DKO cells.

### RSK1/2 are S6-S235/236 kinases in melanoma

The RSK family of genes (RSK1-4) are generally considered S6 kinases [20]: RSK1 and RSK2 are capable of phosphorylating S6 at S235/236 but not at S240/244, while RSK3 and RSK4 play negligible roles [22]. To determine the role of RSK1/2 in melanoma S6 phosphorylation, we generated single and double KOs in 1205Lu using CRISPR. Interestingly, in the single RSK1 or RSK2 KOs with complete protein loss, we saw a ~50% decrease in pS6-S235/236, but no effect in double RSK1/2 KOs (Fig. 4F), suggesting a possible negative feedback loop when both RSKs are lost. Consistent with previous studies, none of the RSK1/2 KOs had any effect on pS6-S240/244. These results help clarify the role of RSK1/2 in S6 phosphorylation in melanoma.

### The effect of S6K1/2 loss on the proteome

**Fig. 5:**
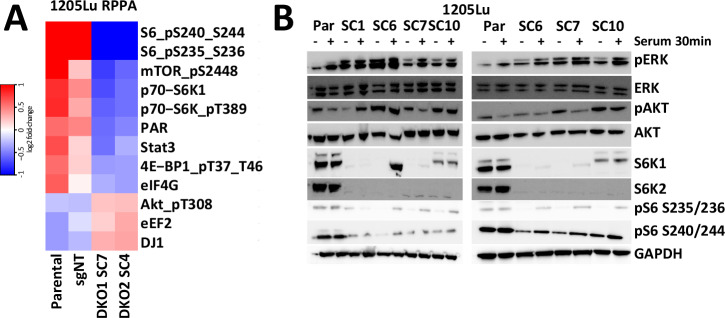
Protein landscape of DKO cells. **A** Top differentially expressed proteins in DKO vs control cells from a reverse phase protein array assay. **B** Western blot in 1205 Lu S6K1/2 DKO cells compared to parental controls, with or without 30 min of serum treatment after 16 h of serum starvation. *Left:* single cell clones from DKO1, r*ight:* single cell clones from DKO2.

To determine the impact of S6K1/2 loss on the proteome, we conducted reverse-phase protein array (RPPA) analysis for 497 antibodies on the 1205Lu DKO cell lines. Compared to parental or non-targeting sgRNAs (sgNT), the DKO lines confirmed pS6, S6K1, and pS6K1 as among the steepest decreases (Fig. 5A), consistent with our western blot data. We also found decreases in p-mTOR, p4EBP1, and eIF4G, and increases in pAKT and eEF2. Western blot analysis confirmed an increase in pAKT in the majority of single-cell-cloned DKO or S6K2 KO cells (Fig. 5B). As pERK levels are not reliably assessed by RPPA, we also performed western blotting for pERK and found that it was consistently upregulated in our DKO cells (Fig. 5B). In total, our data suggest that loss of S6K1/2 disrupts feedback pathways to both AKT/mTOR and MAPK signaling.

### Phosphomimetic S6 overexpression does not confer BRAFi resistance

**Fig. 6:**
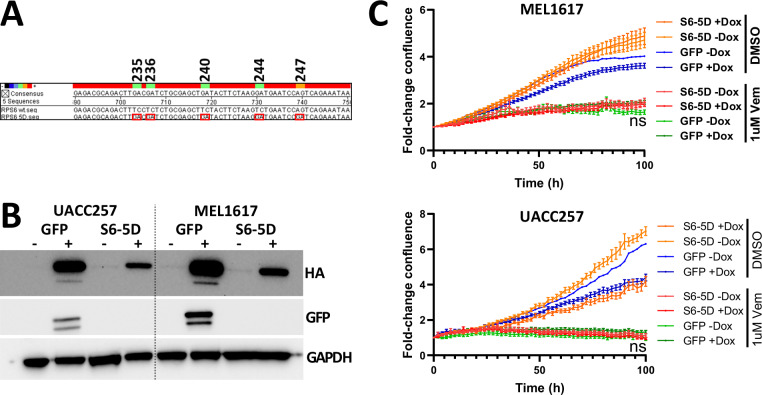
Phosphomimetic S6 does is not sufficient to induce BRAFi resistance. **A** Sanger sequencing results of the S6-5D ORF compared to wild type S6, showing mutation of the 5 serines to aspartic acids. **B** Western blot for BRAFi-sensitive cell lines inducibly expressing HA-tagged phosphomimetic S6 (~32 kDa), with or without dox. The inducible GFP is also HA-tagged (~27 kDa). **C** BRAFi response assay for GFP- and phosphomimetic S6-5D-expressing cells, with or without dox. ns not significant.

We next considered that although S6K1/2 loss did not affect intrinsic BRAFi resistance, perhaps pS6 itself might play a role. As knockout or knockdown of S6 is pan-cell lethal, we instead generated a dox-inducible vector containing a phosphomimetic S6 construct where all 5 major serines (235, 236, 240, 244, and 247) were replaced with aspartic acid (Fig. 6A), which we term S6-5D. We confirmed expression through western blot of the HA-tagged proteins (Fig. 6B). Despite robust expression levels of S6-5D, it had no effect on BRAFi sensitivity in the two BRAFi-sensitive cells lines MEL1617 and UACC257 (Fig. 6C), even when BRAFi was lowered to 100 nM. Therefore, neither S6K1/2 nor pS6 itself appear to play a significant functional role in BRAFi resistance.

### A CRISPR kinome knockout screen identifies FGFR1 as a dependency in intrinsically resistant melanomas

**Fig. 7:**
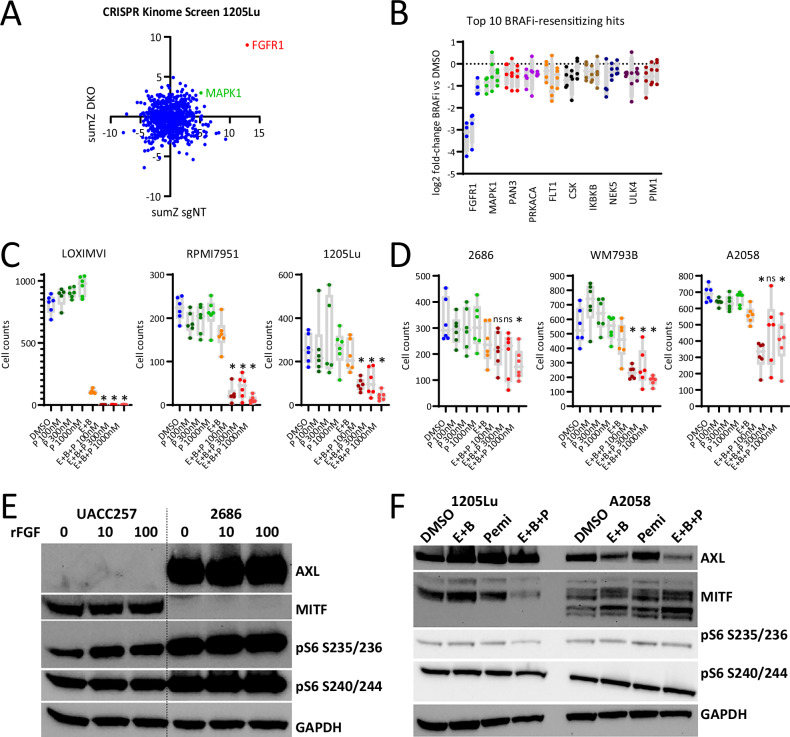
A CRISPR kinome screen identifies FGFR1 as a driver of BRAFi intrinsic resistance in a subset of melanoma cell lines. **A** sumZ scores for the Brunello kinome sgRNA library comparing 1205Lu sgNT and DKO cells, for BRAFi versus DMSO. MAPK1 and FGFR1 are highlighted as dependencies whose loss resensitizes the cells to BRAFi. **B** Performance of individual sgRNAs for the top 10 genes whose sgRNAs resensitized 1205Lu cells to BRAFi. Log2 fold-change of normalized sgRNA abundance is shown for BRAFi-treated vs DMSO-treated cells for 4 sgRNAs per gene. For each gene, from left to right are the results for parental, DKO1, and DKO2 cells. **C**, **D** Drug response assays at 96 h of 6 intrinsically BRAFi-resistant cell lines to treatment with DMSO, BRAFi+MEKi (encorafenib+binimetinib = E + B, both at 300 nM for all treatments), FGFRi (pemigatinib = P), or their combination at the indicated doses. **C** cell lines with a stronger response, **D** cell lines with a more modest response. *p < 0.05 when compared to E + B alone, Student’s *t* test. ns not significant. **E** Western blot in the sensitive line UACC257 and the resistant line 2686 treated with 300 nM encorafenib + 300 nM binimetinib, in the absence or presence of exogenous recombinant FGF1 at the given nM concentrations for 96 h. **F** Western blot in the resistant lines 1205Lu or A2058 in the absence or presence of DMSO, 300 nM encorafenib + 300 nM binimetinib (E + B), 300 nM pemigatinib (Pemi), or the triple combination (E + B + P) for 96 h.

Finally, we sought to unbiasedly identify other kinases whose inhibition might reverse BRAFi resistance, as, to our knowledge, no CRISPR screens have yet been conducted in intrinsically BRAFi-resistant melanoma, only in the setting of acquired resistance [23]. Therefore, using 1205Lu either with or without S6K1/2 DKO, we conducted a pooled CRISPR knockout screen against 763 kinases in the presence or absence of BRAFi. We obtained similar results among all three cell lines (1 parental and 2 DKOs), indicating that S6K1/2 loss did not substantially alter the sensitivity to kinase losses (Supplementary Table 2). The top two kinases that potentially reverse BRAFi resistance were MAPK1, which encodes ERK2 and thus serves as a positive control, and FGFR1 (but not FGFR2/3/4), which was far and away the strongest hit (Fig. 7A, B).

We therefore asked whether the pan-FGFR inhibitor pemigatinib could reverse intrinsic BRAFi resistance. We obtained 3 additional intrinsically resistant cell lines, WM793b, LOXI-MVI, and A2058 (Supplementary Table 1), to supplement our original 3 lines and confirmed their resistant status (Supplementary Fig. 2). Out of these 6 total intrinsically resistant cell lines, 3 showed potent reversal of resistance upon addition of pemigatinib to BRAFi+MEKi: LOXI-MVI, RPMI-7951, and 1205Lu (Fig. 7C). Additionally, the other 3 lines 2686, WM793B, and A2058 showed more modest but still significant resistance reversal, except at lower doses for 2686 and at 300 nM for A2058 (Fig. 7D).

Given the known association of intrinsic resistance with AXL-hi/MITF-low status, we asked if FGFR1 signaling might regulate this state. However, the addition of the FGFR1 ligand FGF1 did not alter AXL/MITF protein levels in either the sensitive line UACC257 or the resistant line 2686 (Fig. 7E), while treatment with pemigatinib on top of BRAFi+MEKi had variable effects, increasing AXL and decreasing MITF in 1205Lu but having the opposite effect in A2058 (Fig. 7F). Interestingly, FGFRi did not decrease pS6 either alone or in combination with BRAFi+MEKi in either cell line. Taken together, these data nominate FGFR1 as a therapeutic vulnerability in intrinsically BRAFi-resistant melanoma, despite it not being a primary regulator of AXL/MITF status.

## Discussion

In this study, we provide the first comprehensive full knockout assessment of S6K1 and/or S6K2 in melanoma, demonstrating that despite the well-established role of changes in S6 phosphorylation as a top marker of intrinsic BRAFi resistance, neither kinase plays a direct functional role in maintaining it. Moreover, overexpression of S6K1, S6K2, both together, or phosphomimetic S6 failed to induce BRAFi resistance, though it should be noted that phosphomimetics do not always fully recapitulate the function of their endogenously phosphorylated counterparts. Instead of the S6K/S6 axis, our kinome-wide CRISPR screen uncovered FGFR1 as a major driver of intrinsic BRAFi resistance and represents a promising counter-resistance target for this understudied patient population.

A previous study identified FGFR1 as a therapeutic vulnerability in acquired BRAFi resistant melanoma [24] but did not assess its role in intrinsic BRAFi resistance. Therefore, this and the present study complement one other, together nominating FGFR1 as a candidate pan-BRAFi resistance target in melanoma. This finding is especially intriguing because the mechanisms of acquired and intrinsic resistance differ greatly, with the former being driven more by secondary MAPK alterations and the latter more by PI3K alterations and/or AXL-high/MITF-low status. Nevertheless, FGFR1 appears to be critical for both. Therefore, our findings theoretically expand the number of patients who might benefit from combined BRAFi+MEKi and FGFRi.

A recent study also functionally assessed S6K1 and S6K2 in NRAS-mutant melanoma but not BRAF-mutant melanoma [25]. Intriguingly, they found that S6K2 (but not S6K1) knockdown by shRNA led to widespread apoptosis in MEKi-resistant NRAS-mutant melanoma, driven by changes in Peroxisome Proliferator-Activated Receptor alpha (PPARa). This result contrasts greatly with our finding that complete S6K2 loss by CRISPR had no effect on BRAF-mutant melanoma viability. Indeed, we observed no decrease in cell number during the first week of S6K2 sgRNA transduction (not shown), likely ruling out an initial cell death followed by stable survival of subclones. Instead, the disparity in our findings might be attributable to innate differences in BRAF- versus NRAS-mutant melanoma, and/or to differences in the phenotypic impacts of shRNA-induced KD versus CRISPR-induced KO. Reconciling these findings will require additional functional work.

In addition to our S6K1/2 and FGFR1-specific findings, we also shed additional light on the regulation of pS6 in melanoma. First, we show that S6K1/2 are not the only S6 kinases, as residual pS6 remains after complete S6K1/2 loss. This residual pS6 is insensitive to rapamycin, indicating the presence of non-mTOR-regulated S6 kinases. Second, we showed that CK1a and RSK1/2 are functional S6 kinases in melanoma. For CK1a, its loss induced a partial decrease in residual pS6 in the absence of S6K1/2, suggesting that it is at least partially responsible for maintaining that residual S6 phosphorylation. For RSK1/2, loss of either protein in parental cells produced a decrease in pS6-S235/236 but had no effect on S240/244, consistent with a report in HEK293E cells [22]. Interestingly, double loss of RSK1/2 in a glioblastoma cell line had minimal effects on pS6-S235/236, possibly indicating cell type specificity [26]. The identity of any other kinase(s) responsible for the remaining residual S6 phosphorylation in S6K1/2 DKO cells, especially of S240/244, which had consistently higher residual levels than S235/236, will be of interest to discover. Finally, to our knowledge, our study documents the first exogenous overexpression of phosphomimetic S6 in any cancer setting. Despite a lack of effect on BRAFi sensitivity, this construct will be of broad utility to the research field, as pS6 plays many roles in cancer in addition to drug resistance.

In conclusion, our results comprehensively show that S6K1/2 are not primary drivers of intrinsic BRAFi resistance in melanoma, indicating that therapeutic research would be better redirected towards more promising targets such as FGFR1. These findings may also have implications for the role of S6K1/2 in intrinsic TKI resistance in other cancer types as well, such as lung and colon cancer. Excitingly, however, the FGFR inhibitors pemigatinib (used in this study), infigratinib, and futibatinib have been granted FDA approval for use in FGFR2 fusion-positive cholangiocarcinoma and erdafitinib in FGFR3-mutant bladder cancer, opening the door for FGFRi use in other cancers. In total, our findings help clarify the translational landscape of intrinsic BRAFi resistance in melanoma and nominate FGFR1 as a promising new target.

## Methods

### Plasmids and viral infections

In order to overexpress S6K1 and S6K2, we first purchased Gateway-compatible donor ORF plasmids from Addgene, respectively: R777-E297 Hs.RPS6KB1 (#70581, which encodes the p85 isoform of S6K1) and pDonor233-RPS6KB2 (#23533). To generate the p70 isoform of S6K1, PCR was performed with the forward primer 5’-ATGGCAGGAGTGTTTGACATAGA-3’ and the reverse primer: 5’-TTATAGATTCATACGCAGGTGCTC -3’, using R777-E297 Hs.RPS6KB1 as the template. These 3 donor vectors were then recombined into the dox-inducible backbones pLIX-403 (#41395) or pInducer20 (#44012) using LR Clonase II Enzyme mix (Life Technology, Cat. 11791020) to generate the final overexpression vectors pLix403-S6KB1-p85-puro, pLix403-S6KB1-p70-puro, and pInducer20-S6KB2-neo. The S6K1 and S6K2 pLentiCRISPRv2 lentiviral sgRNAs contructs were purchased from Genscript while shRNAs were purchased from Sigma. All sgRNA and shRNA details can be found in Supplementary Table 3.

To generate the phosphomimetic S6-5D construct, we first designed substitution mutations to convert the serines at 235, 236, 240, 244, and 247 into aspartic acid. This was then purchased as a synthesized construct in a Gateway-compatible donor vector (Genscript). An N-terminal HA-tag was added by PCR and the ORF was cloned into pLIX-403 via LR Clonase II to generate pLIX403-RPS6-5D.

Lentivirus for all constructs was produced by transfecting HEK 293T cells with the expression plasmids and the packaging vectors pVSVG and psPAX, using PEI (3 μg per μg of DNA). Viral supernatants were collected after 72 h, filtered through a 0.45-μm filter and used to infect target cells in addition to polybrene (8 μg/mL, Millipore). After 24 h, the media was changed and the cells were placed under antibiotic selection (puromycin 2 μg/mL, and/or hygromycin 100 ug/mL, and/or G418 1000 ug/mL).

### Cell culture

Cell lines were cultured in RPMI 1640 (Life Technology #11875189), DMEM/F12 (ThermoFisher #11320082) or DMEM (Life Technology #11995073) containing 10% (v/v) FBS (Thermo Fisher Scientific, 26140079) and 1% (v/v) penicillin/streptomycin (Thermo Fisher Scientific, 15-140-122) in an incubator at 37 °C with 5% CO_2_, according to the requirements of each line. Doxycycline was consistently used at a concentration of 2ug/ml to induce gene expression from pLIX403 and pInducer20 constructs. All cell lines were obtained from other labs, whose original source was either the Wistar Institute or ATCC and were STR-verified and tested myco negative.

### Antibodies and Western blot

For western blots, cells were lysed in RIPA butter (Teknova # R3792) with protease inhibitors tablet (Pierce # A32959), total protein were quantified by Bradford (Pierce # PI23236), and then 30 ug protein/lane was loaded in a 4–12% gradient SDS-PAGE gel (Invitrogen # NW04125BOX). After electrophoresis, total proteins were transferred onto a nitrocellulose membrane (BioRAD #1704271) using a BioRAD Trans-Blot Turbo Transfer System (BioRAD #1704150). Membranes were blocked in TBS-T buffer (20 mM Tris base, 150 mM NaCl, 0.05% Tween 20, pH 7.4) containing 5% nonfat milk (BioRAD #1706404) at room temperature for 2 h prior to the addition of primary antibody and incubated at 4 °C overnight. Primary antibodies were diluted in TBST plus 5% nonfat milk, while phospho-primary antibodies were diluted in TBST plus 5% BSA. All antibody details can be found in Supplementary Table 4. 3×10 min of TBST–washed membranes were incubated with the goat anti-rabbit or goat anti-mouse secondary antibodies in TBST plus 5% nonfat milk and then washed with TBST (3×10 min). The chemiluminescence was detected by Pierce™ SuperSignal™ West Pico PLUS Chemiluminescent Substrate (Pierce #PI34580) and was then visualized using a ChemiDoc Imaging System (BioRAD #12003153). In some cases, the cells were starved of fetal bovine serum for 16 h, then serum was added back in 30 min prior to protein isolation (“Serum 30 min”). This was done because S6 phosphorylation is responsive to serum starvation and add-back can therefore serve as an additional internal control for particularly sensitive treatments. Western blots were quantitated on ImageJ by subtracting out the background and normalizing to GAPDH.

### Inhibitors and drug assays

All inhibitors were purchased commercially, and details can be found in Supplementary Table 5. For drug assays, 5000 cells were cultured in each well of a 48-well plate or 1000 cells in each well of a 96-well plate, and the appropriate inhibitors were added to the cell culture media to achieve final concentrations. Cell growth was evaluated using the Sartorius Essen IncuCyte ZOOM Live Cell Analyzer or by standard staining with crystal violet, dissolution with 10% acetic acid, and reading in a plate reader at OD590. For mechanistic experiments, we chose to use BRAFi alone to focus on BRAF-specific downstream effects, while for efficacy experiments, we primarily chose to use BRAFi+MEKi to provide stronger clinical relevance.

### CRISPR Kinome screen

We purchased the Brunello pooled human kinome CRISPR knockout library (Addgene #75314), containing 4 guides for each of 763 human kinases, totaling 3,052 unique sgRNAs. In 15-cm dishes, 10 million 1205Lu cells (sgNT, DKO1 SC7, or DKO2 SC4) were transduced using a multiplicity of infection (MOI) of 0.3. Since DKO cells already contain puromycin resistance (from pLentiCRISPRv2-puro), we could not select in puromycin; therefore, we instead used 5× the number of cells normally used for such a screen and ensured high numbers of cells at each passage so as not to dilute or lose sgRNA-containing cells. Each cell line was then divided into two groups, either treated with vehicle (0.01% DMSO) or 1 μM Vemurafenib. Cells were passaged every three days, and genomic DNA was collected at the end of treatment. Sequencing libraries were created by purifying PCR products to eliminate leftover primers and amplification byproducts, indexing them with sample-specific barcodes, and pooling them. Prior to next-generation sequencing, libraries were evaluated for fragment size distribution and concentration to guarantee consistent representation and sequencing quality. After demultiplexing and aligning sequencing data to the reference sgRNA library, the relative enrichment or depletion of particular sgRNAs was quantified and ranked using the DrugZ algorithm [27].

### RPPA

The RPPA data analysis was performed according to the procedure from the M.D. Anderson Cancer Center. 1205Lu (Parental, sgNT, DKO1 SC7, and DKO2 SC4) cell lines were washed with cold PBS and lysed with RPPA lysis buffer. The supernatants were collected after centrifugation and quantified using a Bradford assay. Samples were submitted to the RPPA core facility and processed according to previously described protocols [16].

### Statistics

Prism 9 software was used to analyze and present quantitative data with the format of mean ± SEM. *P* < 0.05 is considered significant using Student’s *T* tests or ANOVA, all two-sided. All experiments were run in at least triplicate.

## Supplementary information

Supplemental Figure 1

Supplemental Figure 2

Uncropped western blots

Supplemental Tables

Supplemental Figure legend

## Data Availability

All relevant data are included in this article and its supplementary materials. Additional information is available from the corresponding author upon reasonable request.

## References

[1] Corcoran RB, Rothenberg SM, Hata AN, Faber AC, Piris A, Nazarian RM, et al. TORC1 suppression predicts responsiveness to RAF and MEK inhibition in BRAF-mutant melanoma. Sci Transl Med. 2013;5:196ra198. 10.1126/scitranslmed.3005753 PMC3867020 23903755

[2] Robert C, Grob JJ, Stroyakovskiy D, Karaszewska B, Hauschild A, Levchenko E, et al. Five-year outcomes with dabrafenib plus trametinib in metastatic melanoma. N Engl J Med. 2019;381:626–36. 31166680 10.1056/NEJMoa1904059

[3] Ugurel S, Rohmel J, Ascierto PA, Becker JC, Flaherty KT, Grob JJ, et al. Survival of patients with advanced metastatic melanoma: the impact of MAP kinase pathway inhibition and immune checkpoint inhibition - update 2019. Eur J Cancer. 2020;130:126–38. 32179447 10.1016/j.ejca.2020.02.021

[4] Khalaileh A, Dreazen A, Khatib A, Apel R, Swisa A, Kidess-Bassir N, et al. Phosphorylation of ribosomal protein S6 attenuates DNA damage and tumor suppression during development of pancreatic cancer. Cancer Res. 2013;73:1811–20. 23361300 10.1158/0008-5472.CAN-12-2014

[5] Grasso S, Tristante E, Saceda M, Carbonell P, Mayor-Lopez L, Carballo-Santana M, et al. Resistance to Selumetinib (AZD6244) in colorectal cancer cell lines is mediated by p70S6K and RPS6 activation. Neoplasia. 2014;16:845–60. 25379021 10.1016/j.neo.2014.08.011 PMC4212257

[6] Hirashita Y, Tsukamoto Y, Kudo Y, Kakisako D, Kurogi S, Hijiya N, et al. Early response in phosphorylation of ribosomal protein S6 is associated with sensitivity to trametinib in colorectal cancer cells. Lab Invest. 2021;101:1036–47. 33911189 10.1038/s41374-021-00590-w

[7] Yang-Kolodji G, Mumenthaler SM, Mehta A, Ji L, Tripathy D. Phosphorylated ribosomal S6 (p-rpS6) as a post-treatment indicator of HER2 signalling targeted drug resistance. Biomarkers. 2015;20:313–22. 26329528 10.3109/1354750X.2015.1068865 PMC7447531

[8] Wang B, Zhang W, Zhang G, Kwong L, Lu H, Tan J, et al. Targeting mTOR signaling overcomes acquired resistance to combined BRAF and MEK inhibition in BRAF-mutant melanoma. Oncogene. 2021;40:5590–9. 34304249 10.1038/s41388-021-01911-5 PMC8445818

[9] Lou K, Steri V, Ge AY, Hwang YC, Yogodzinski CH, Shkedi AR, et al. KRASG12C inhibition produces a driver-limited state revealing collateral dependencies. Sci Signal. 2019;12:eaaw9450. 31138768 10.1126/scisignal.aaw9450 PMC6871662

[10] Ahronian LG, Sennott EM, Van Allen EM, Wagle N, Kwak EL, Faris JE, et al. Clinical acquired resistance to RAF inhibitor combinations in BRAF-mutant colorectal cancer through MAPK pathway alterations. Cancer Discov. 2015;5:358–67. 25673644 10.1158/2159-8290.CD-14-1518 PMC4390490

[11] Rizos H, Menzies AM, Pupo GM, Carlino MS, Fung C, Hyman J, et al. BRAF inhibitor resistance mechanisms in metastatic melanoma: spectrum and clinical impact. Clin Cancer Res. 2014;20:1965–77. 24463458 10.1158/1078-0432.CCR-13-3122

[12] Shi H, Hugo W, Kong X, Hong A, Koya RC, Moriceau G, et al. Acquired resistance and clonal evolution in melanoma during BRAF inhibitor therapy. Cancer Discov. 2014;4:80–93. 24265155 10.1158/2159-8290.CD-13-0642 PMC3936420

[13] Konieczkowski DJ, Johannessen CM, Abudayyeh O, Kim JW, Cooper ZA, Piris A, et al. A melanoma cell state distinction influences sensitivity to MAPK pathway inhibitors. Cancer Discov. 2014;4:816–27. 24771846 10.1158/2159-8290.CD-13-0424 PMC4154497

[14] Müller J, Krijgsman O, Tsoi J, Robert L, Hugo W, Song C et al. Low MITF/AXL ratio predicts early resistance to multiple targeted drugs in melanoma. Nat Commun.2014;5:5712. 10.1038/ncomms6712 PMC4428333 25502142

[15] Boshuizen J, Koopman LA, Krijgsman O, Shahrabi A, van den Heuvel EG, Ligtenberg MA, et al. Cooperative targeting of melanoma heterogeneity with an AXL antibody-drug conjugate and BRAF/MEK inhibitors. Nat Med. 2018;24:203–12. 29334371 10.1038/nm.4472

[16] Romano G, Chen P-L, Song P, McQuade JL, Liang RJ, Liu M et al. A preexisting rare PIK3CAE545K subpopulation confers clinical resistance to MEK plus CDK4/6 inhibition in NRAS melanoma and is dependent on S6K1 signaling. Cancer Discov. 2018;8:556–67. 10.1158/2159-8290.CD-17-0745 PMC5932238 29496665

[17] Shen H, Wang GC, Li X, Ge X, Wang M, Shi ZM, et al. S6K1 blockade overcomes acquired resistance to EGFR-TKIs in non-small cell lung cancer. Oncogene. 2020;39:7181–95. 33037411 10.1038/s41388-020-01497-4 PMC7718330

[18] Zhang Y, Wang Q, Chen L, Yang H-S. Inhibition of p70S6K1 activation by Pdcd4 overcomes the resistance to an IGF-1R/IR inhibitor in colon carcinoma cells. Mol Cancer Therapeutics. 2015;14:799–809. 10.1158/1535-7163.MCT-14-0648 PMC4456303 25573956

[19] Saxton RA, Sabatini DM. mTOR signaling in growth, metabolism, and disease. Cell. 2017;168:960–76. 28283069 10.1016/j.cell.2017.02.004 PMC5394987

[20] Theodosakis N, Micevic G, Langdon CG, Ventura A, Means R, Stern DF, et al. p90RSK blockade inhibits dual BRAF and MEK inhibitor-resistant melanoma by targeting protein synthesis. J Invest Dermatol. 2017;137:2187–96. 28599981 10.1016/j.jid.2016.12.033 PMC6342201

[21] Hutchinson JA, Shanware NP, Chang H, Tibbetts RS. Regulation of ribosomal protein S6 phosphorylation by casein kinase 1 and protein phosphatase 1. J Biol Chem. 2011;286:8688–96. 21233202 10.1074/jbc.M110.141754 PMC3048750

[22] Roux PP, Shahbazian D, Vu H, Holz MK, Cohen MS, Taunton J, et al. RAS/ERK signaling promotes site-specific ribosomal protein S6 phosphorylation via RSK and stimulates cap-dependent translation. J Biol Chem. 2007;282:14056–64. 17360704 10.1074/jbc.M700906200 PMC3618456

[23] Li Z, Wang B, Gu S, Jiang P, Sahu A, Chen CH, et al. CRISPR screens identify essential cell growth mediators in BRAF inhibitor-resistant melanoma. Genom Proteom Bioinforma. 2020;18:26–40. 10.1016/j.gpb.2020.02.002 PMC7393575 32413516

[24] Wang VE, Xue JY, Frederick DT, Cao Y, Lin E, Wilson C, et al. Adaptive resistance to dual BRAF/MEK inhibition in BRAF-driven tumors through autocrine FGFR pathway activation. Clin Cancer Res. 2019;25:7202–17. 31515463 10.1158/1078-0432.CCR-18-2779 PMC6891193

[25] Lipchick B, Guterres AN, Chen HY, Zundell DM, Del Aguila S, Reyes-Uribe PI, et al. Selective abrogation of S6K2 identifies lipid homeostasis as a survival vulnerability in MAPK inhibitor-resistant NRAS-mutant melanoma. Sci Transl Med. 2025;17:eadp8913. 39908352 10.1126/scitranslmed.adp8913 PMC12258192

[26] Roffé M, Nascimento DP, Nunes PB, Soares LC, Alves ADH, Hamraghani A, et al. RSK1 and RSK2 modulate the translatome of glioblastoma cells in an isoform-specific and mTORC1 independent manner. Neurooncol Adv. 2025;7:vdaf144. 41122673 10.1093/noajnl/vdaf144 PMC12536492

[27] Colic M, Wang G, Zimmermann M, Mascall K, McLaughlin M, Bertolet L, et al. Identifying chemogenetic interactions from CRISPR screens with drugZ. Genome Med. 2019;11:52. 31439014 10.1186/s13073-019-0665-3 PMC6706933

