# Supplementary figures and images for: FGFR1 but not S6K1/2 drives intrinsic BRAF inhibitor resistance in melanoma
Source: Cell Death Discov. 2026 May 19;12:294. doi: 10.1038/s41420-026-03155-2 (PMC13350743; doi:10.1038/s41420-026-03155-2)

Supplemental Figure 1

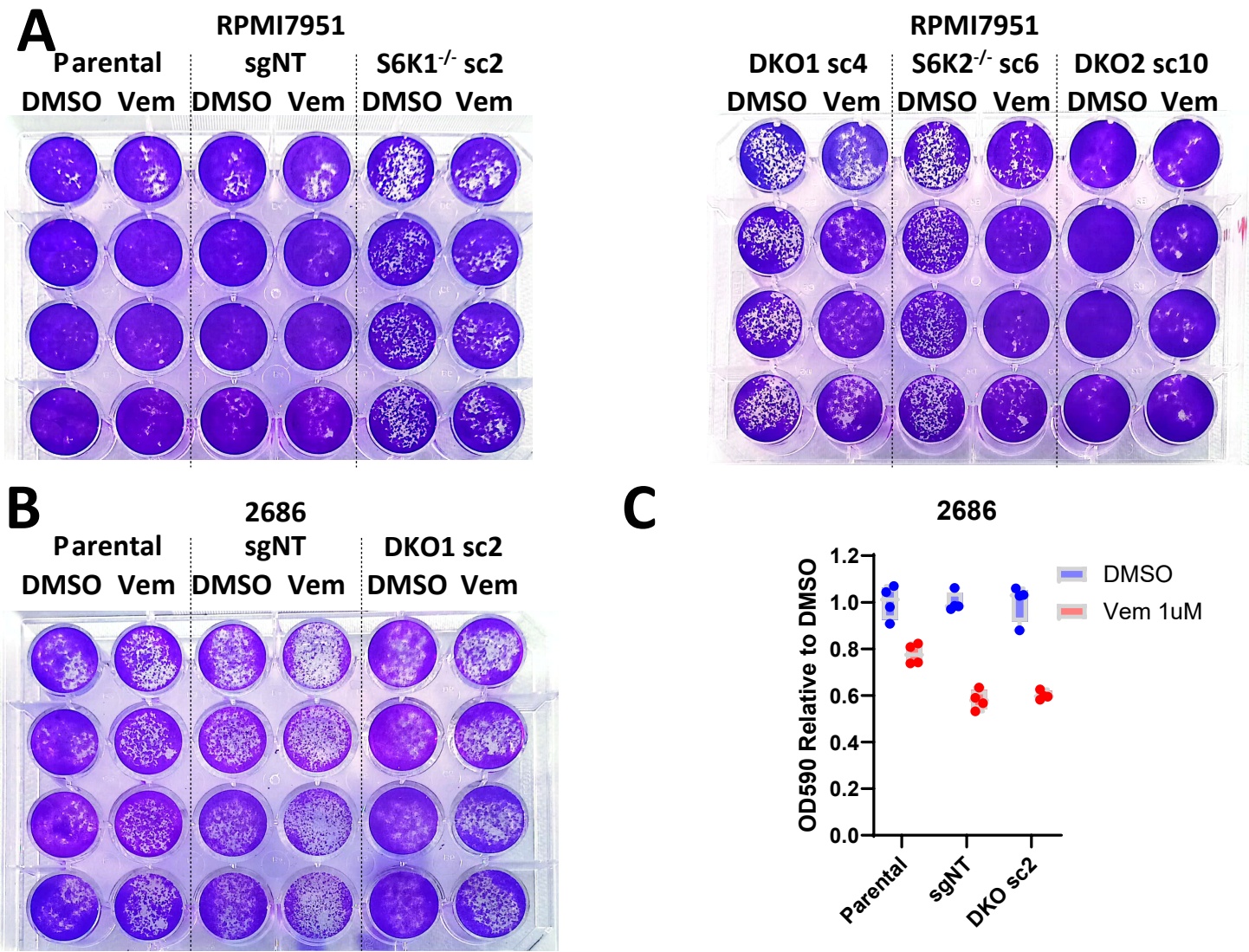

Supplement: Supplementary file 1 — Supplemental Figure 1 [file 41420_2026_3155_MOESM1_ESM.pdf]
