# Supplementary material for: FGFR1 but not S6K1/2 drives intrinsic BRAF inhibitor resistance in melanoma
Source: Cell Death Discov. 2026 May 19;12:294. doi: 10.1038/s41420-026-03155-2 (PMC13350743; doi:10.1038/s41420-026-03155-2)
Supplement: Supplementary file 2 — Supplemental Figure 2 [file 41420_2026_3155_MOESM2_ESM.pdf]

Supplemental Figure 2

Binimetinib

Encorafenib (rows) × Binimetinib (columns)

A2058

0.49	0.86	0.93	0.92	0.95	0.97
0.56	0.86	0.99	1.19	1.28	1.34
0.71	0.95	1.35	1.39	1.48	1.41
0.6	1.08	1.41	1.42	1.42	1.5
0.69	1.13	1.19	1.44	1.41	1.42
0.62	0.93	0.93	0.96	1.04	1

LOXIMV2

0.99	1.19	1.15	1.27	1.39	1.31
1.23	1.34	1.3	1.34	1.46	1.35
1.01	1.29	1.38	1.38	1.36	1.27
1.08	1.1	1.12	1.15	0.65	1.35
0.94	1.17	1.14	1.18	1.19	1.14
0.93	1.01	0.93	0.94	1.01	1

WM793

0.44	0.65	0.82	0.7	0.77	0.82
0.65	0.8	0.7	0.91	0.82	0.94
0.63	0.71	0.95	0.94	0.91	1.02
0.56	0.81	0.76	1.07	0.98	1.1
0.65	0.86	0.96	0.97	1.04	1.16
0.46	0.83	0.8	0.91	0.93	1
