# Supplementary material for: FGFR1 but not S6K1/2 drives intrinsic BRAF inhibitor resistance in melanoma
Source: Cell Death Discov. 2026 May 19;12:294. doi: 10.1038/s41420-026-03155-2 (PMC13350743; doi:10.1038/s41420-026-03155-2)

Figure 1B

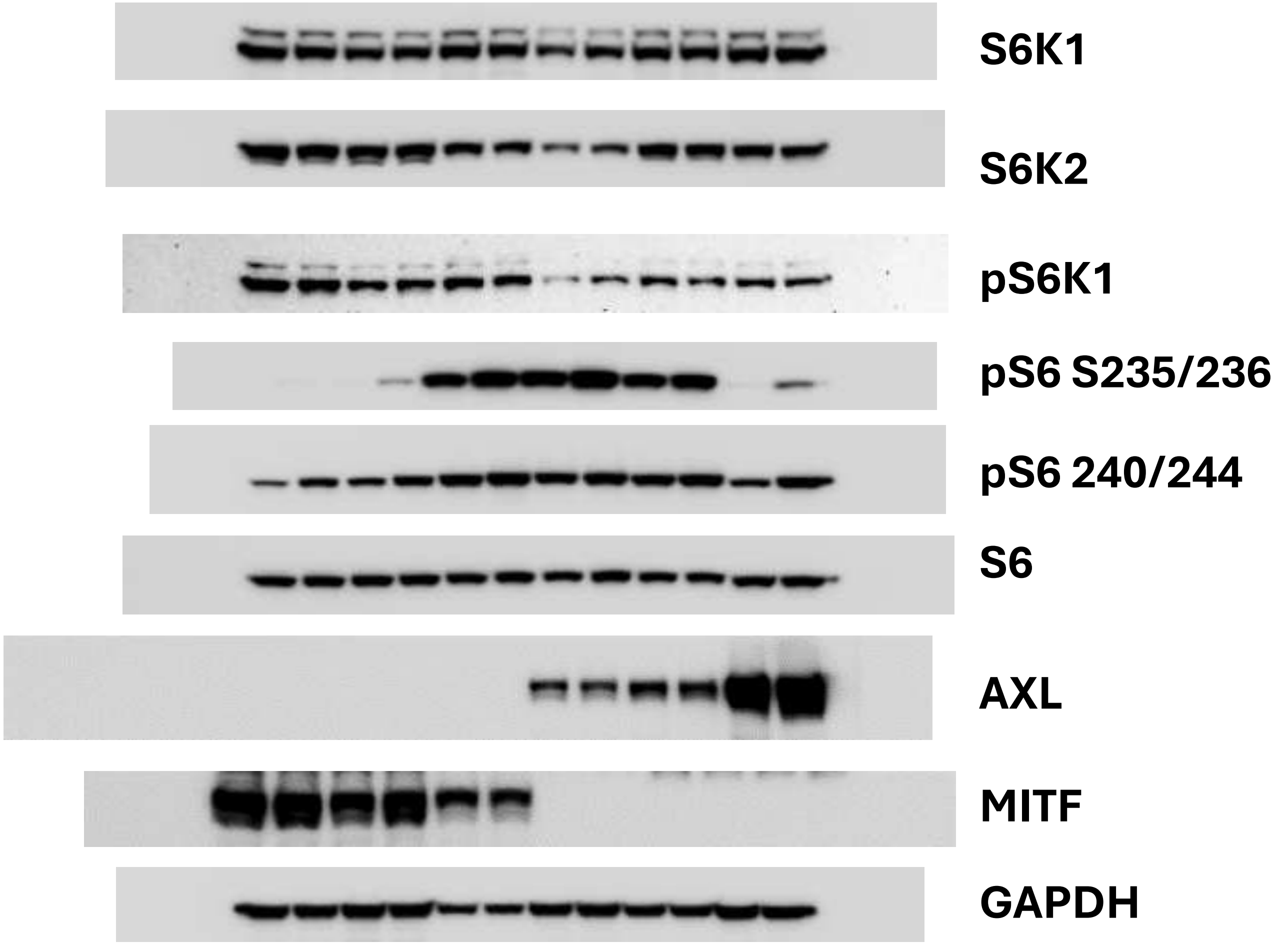

Figure 1C

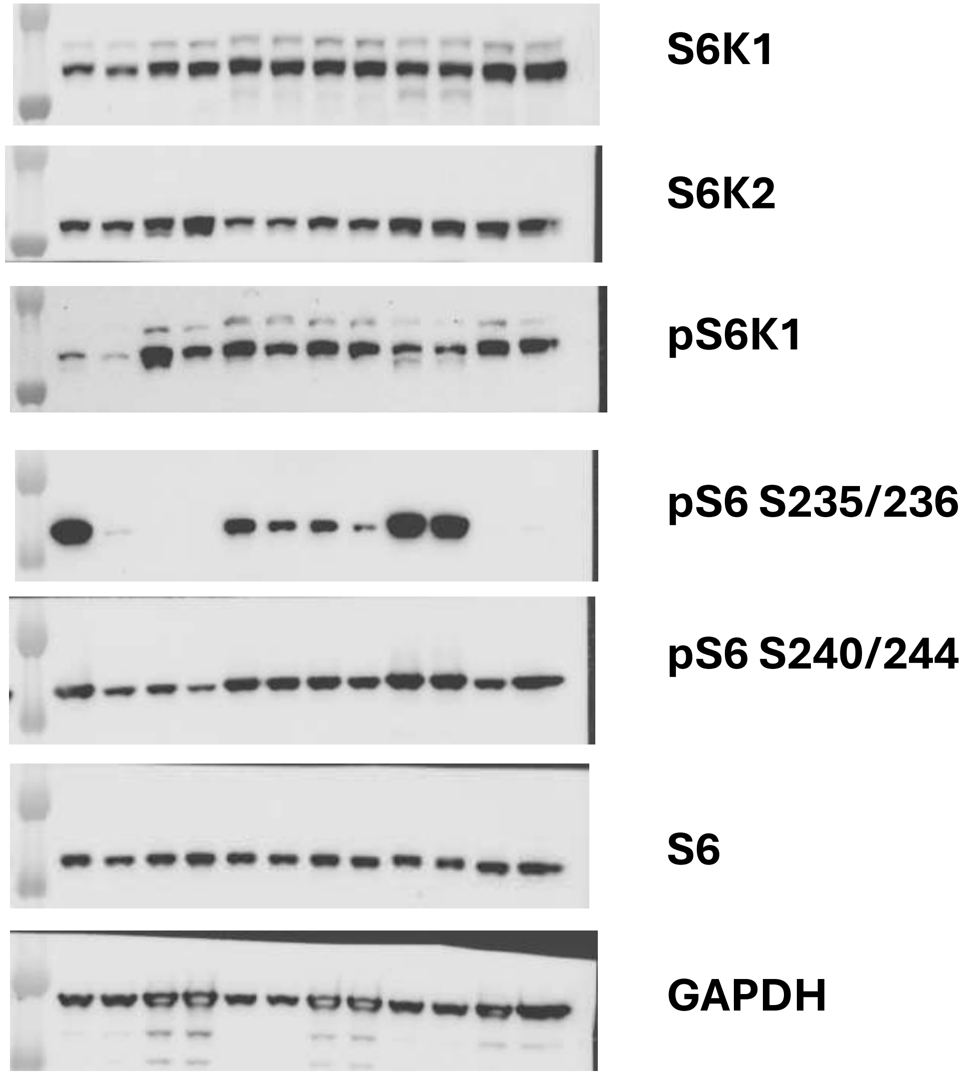

Figure 2A

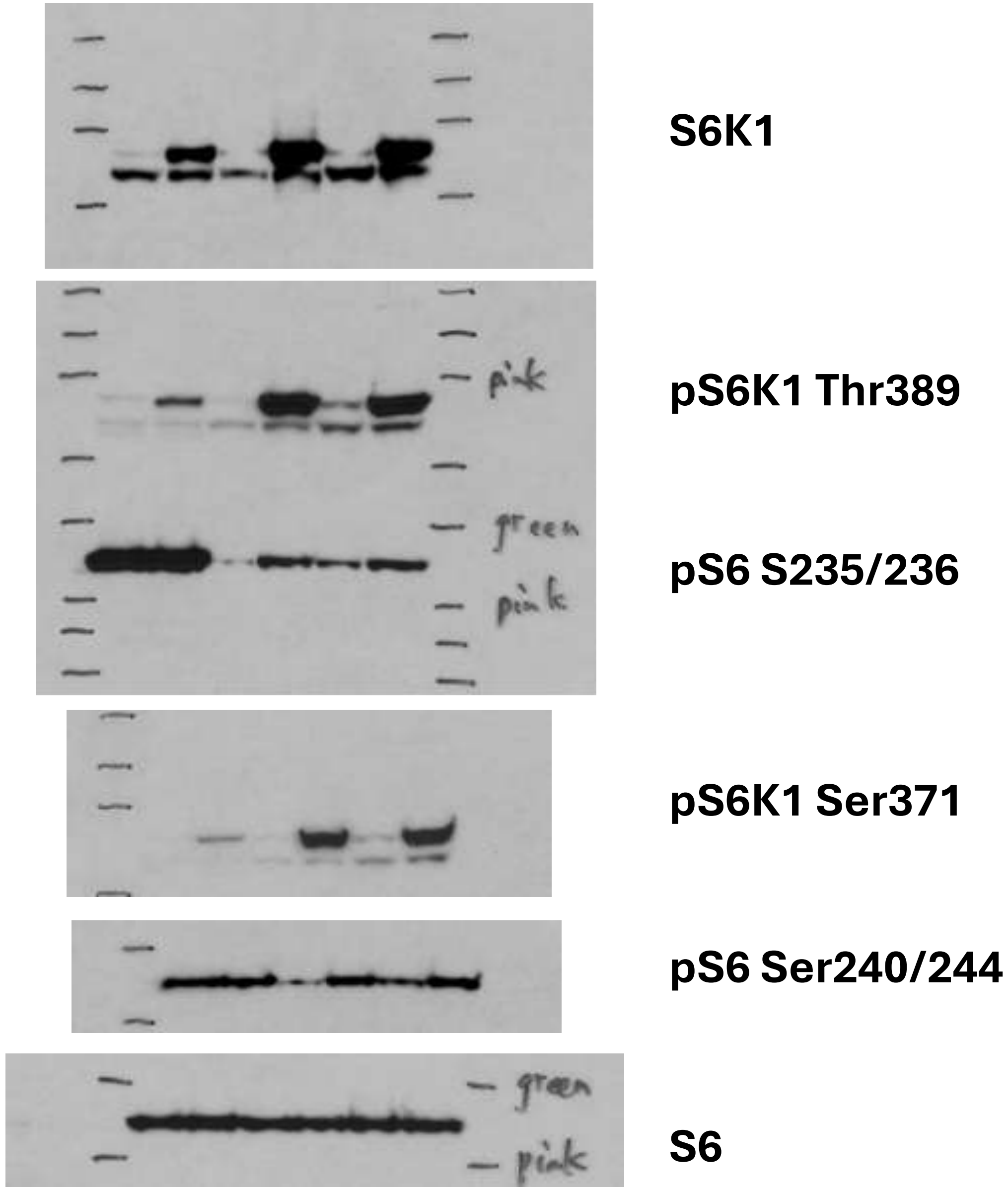

Figure 2B

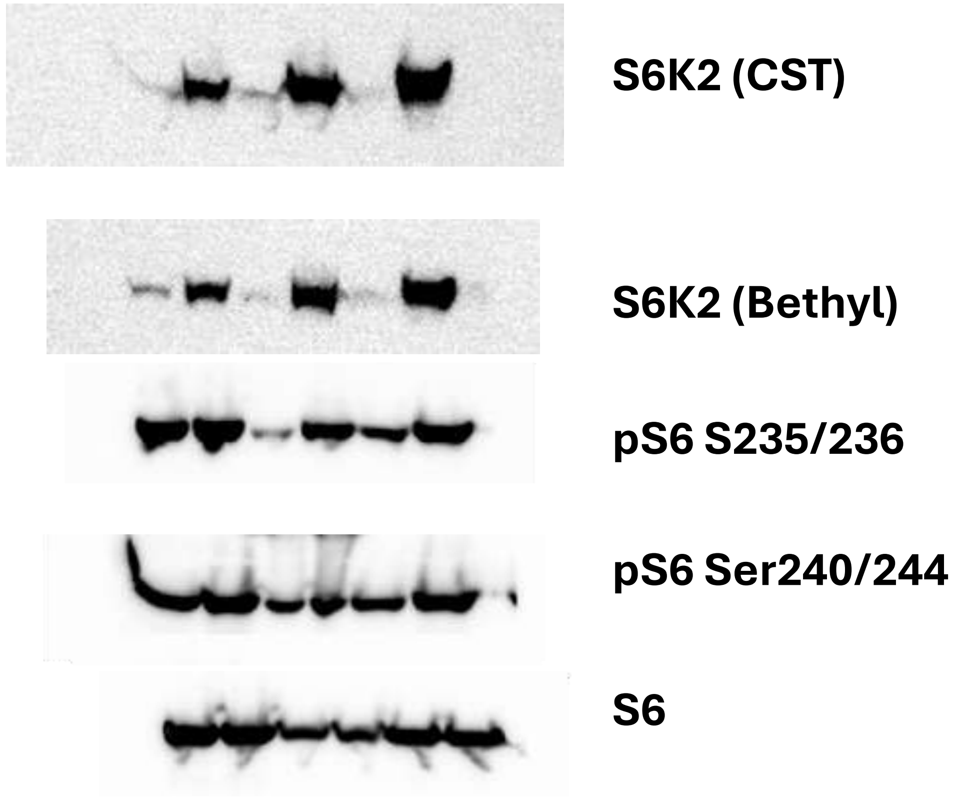

Figure 2C

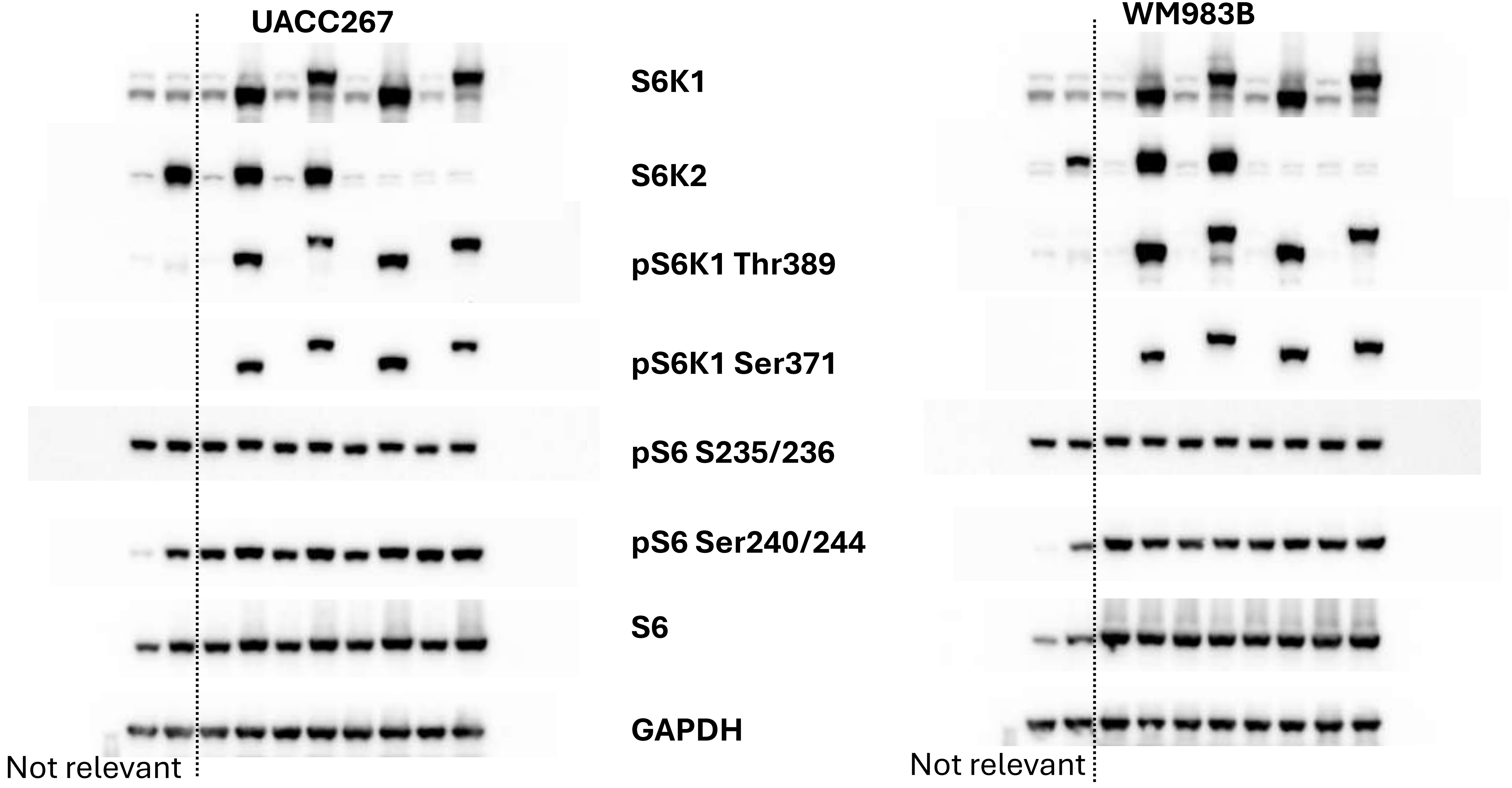
UACC267
WM983B
S6K1
S6K2
pS6K1 Thr389
pS6K1 Ser371
pS6 S235/236
pS6 Ser240/244
S6
GAPDH
Not relevant
Not relevant

Figure 3A

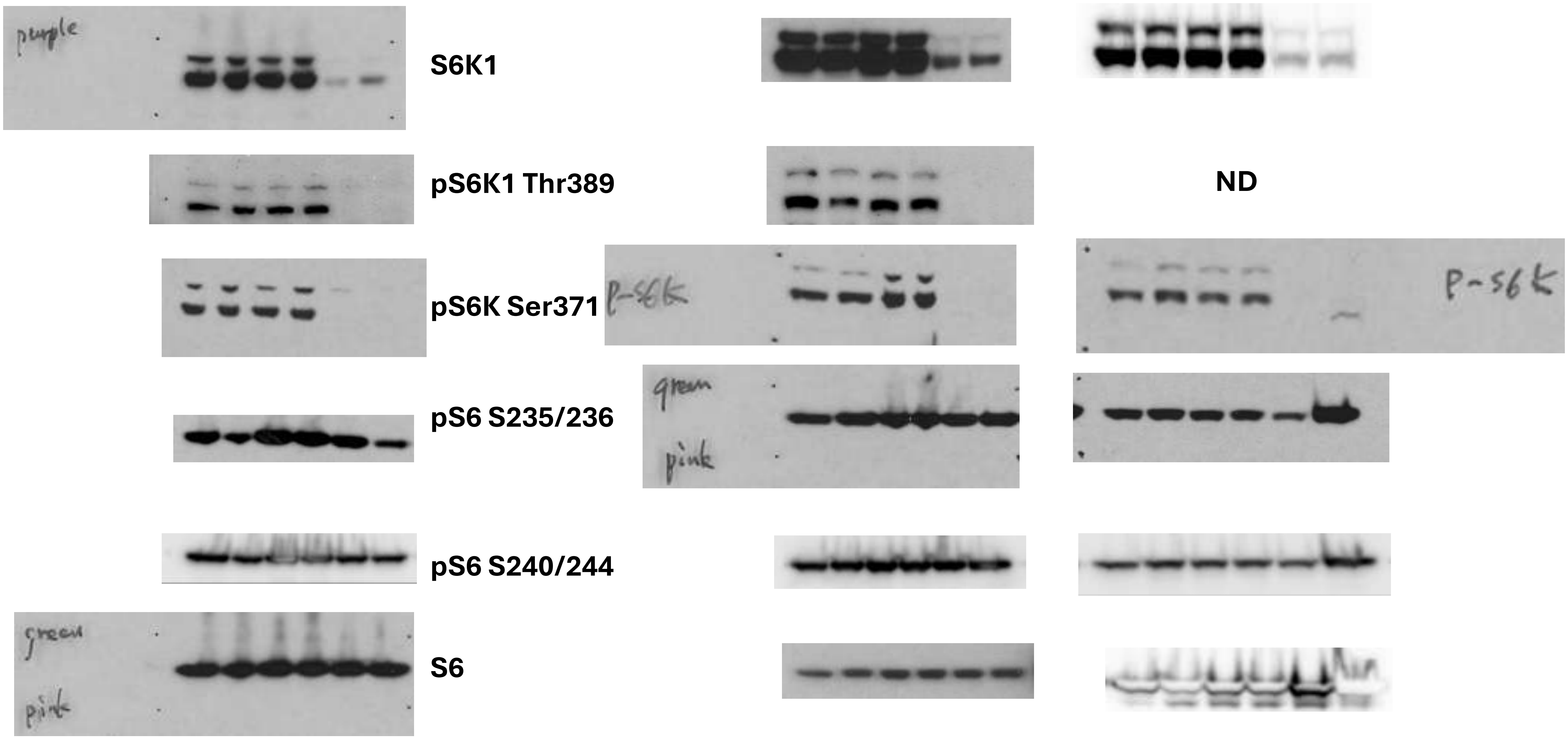

Figure 3B

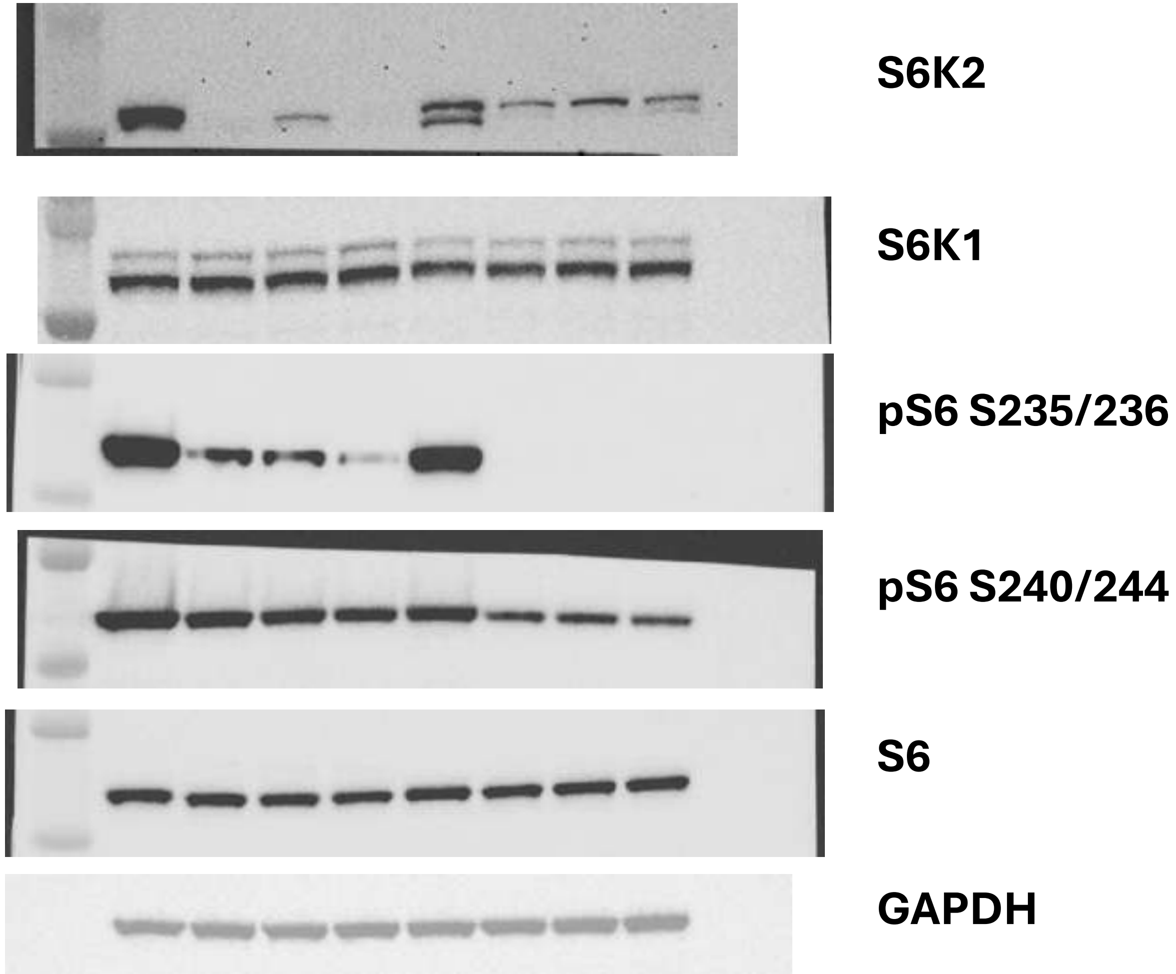

Figure 3C

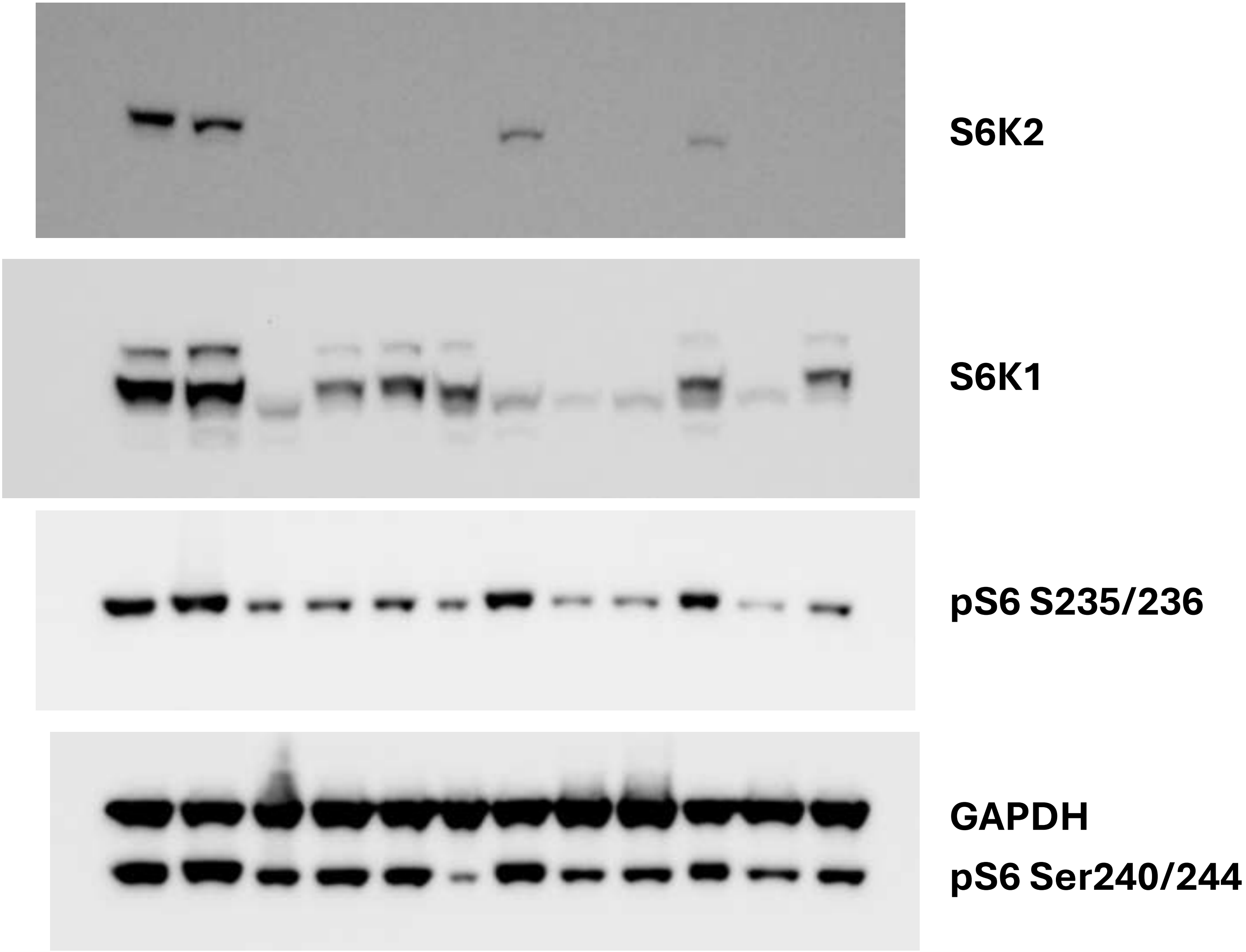

Figure 3D

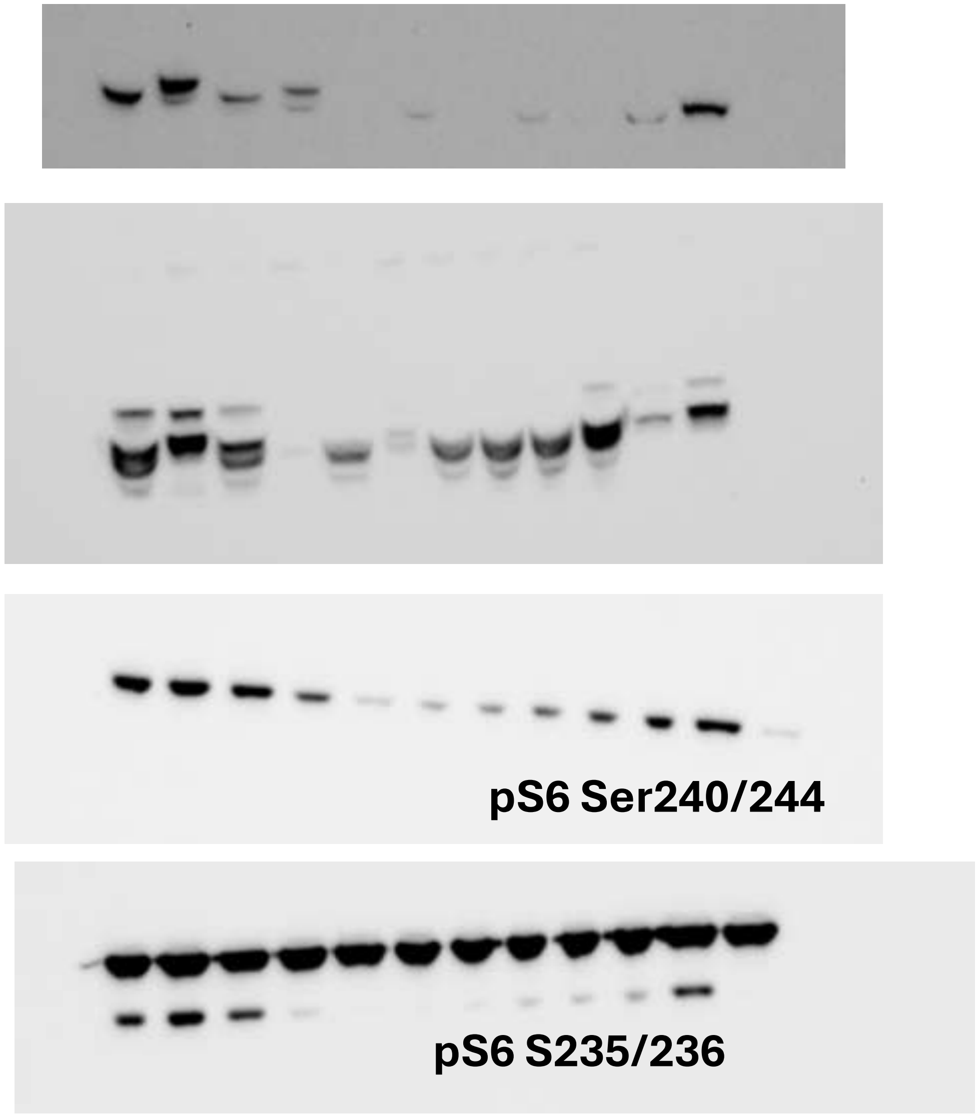

Figure 4A

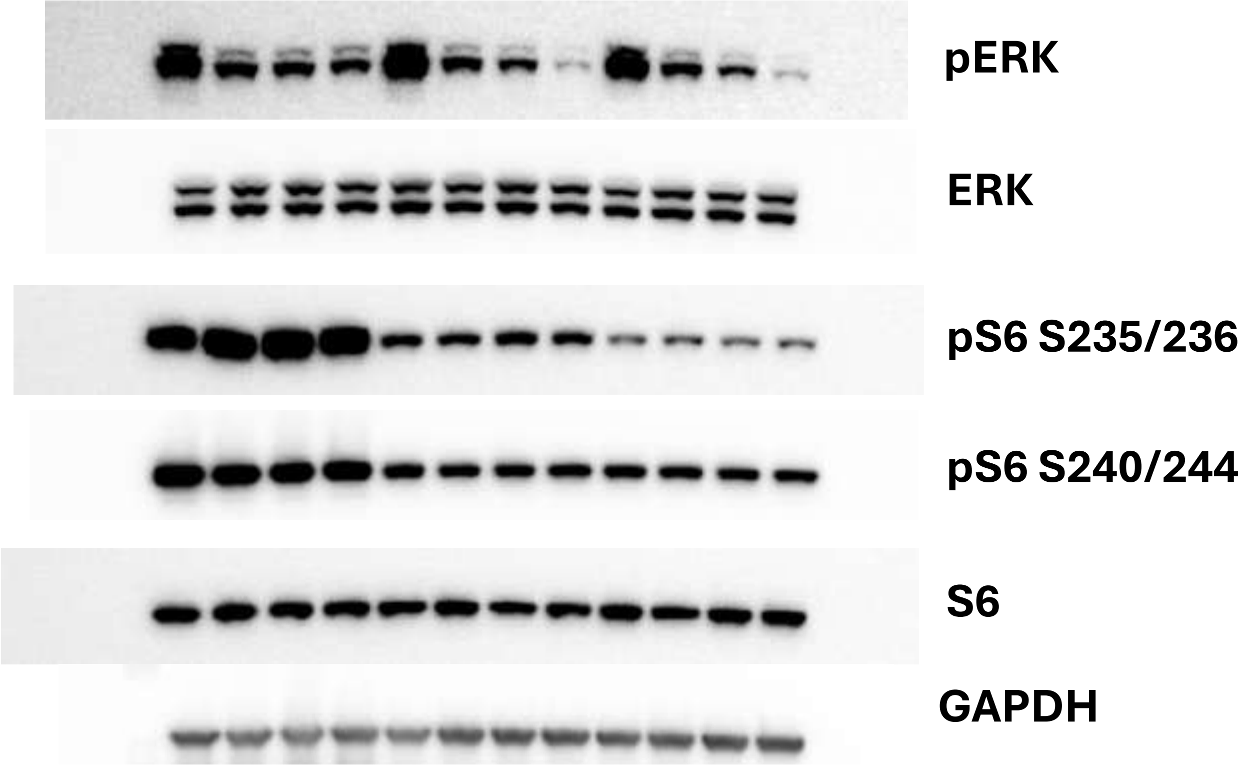

Figure 4B

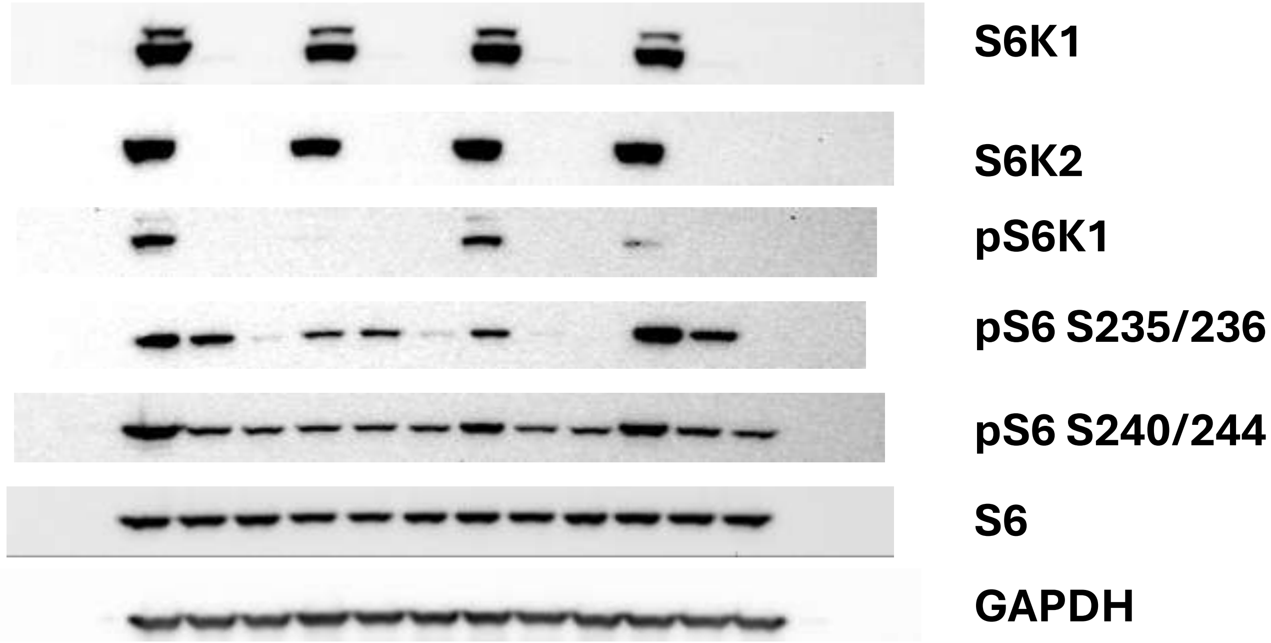

Figure 4D

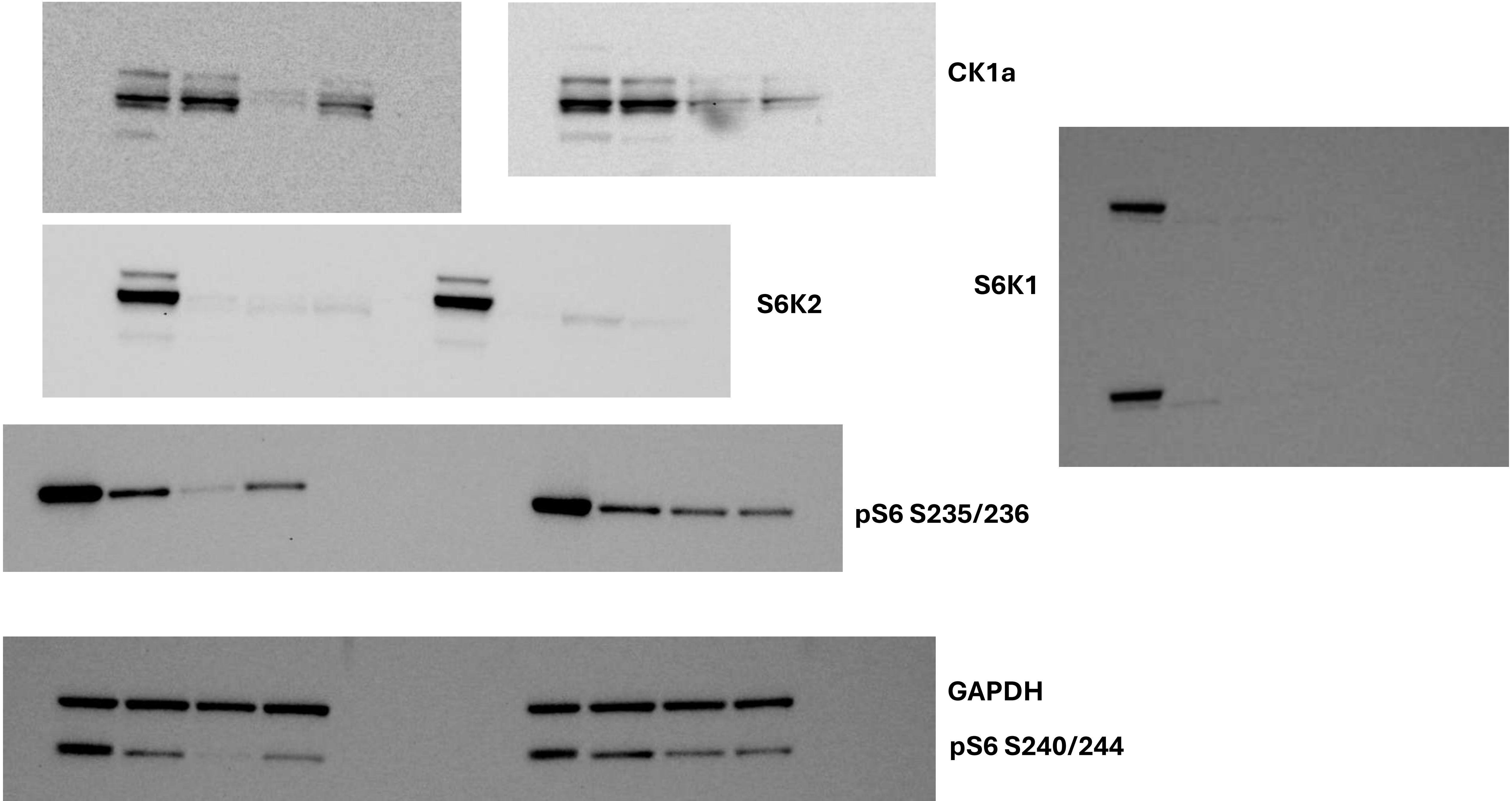

Figure 4F

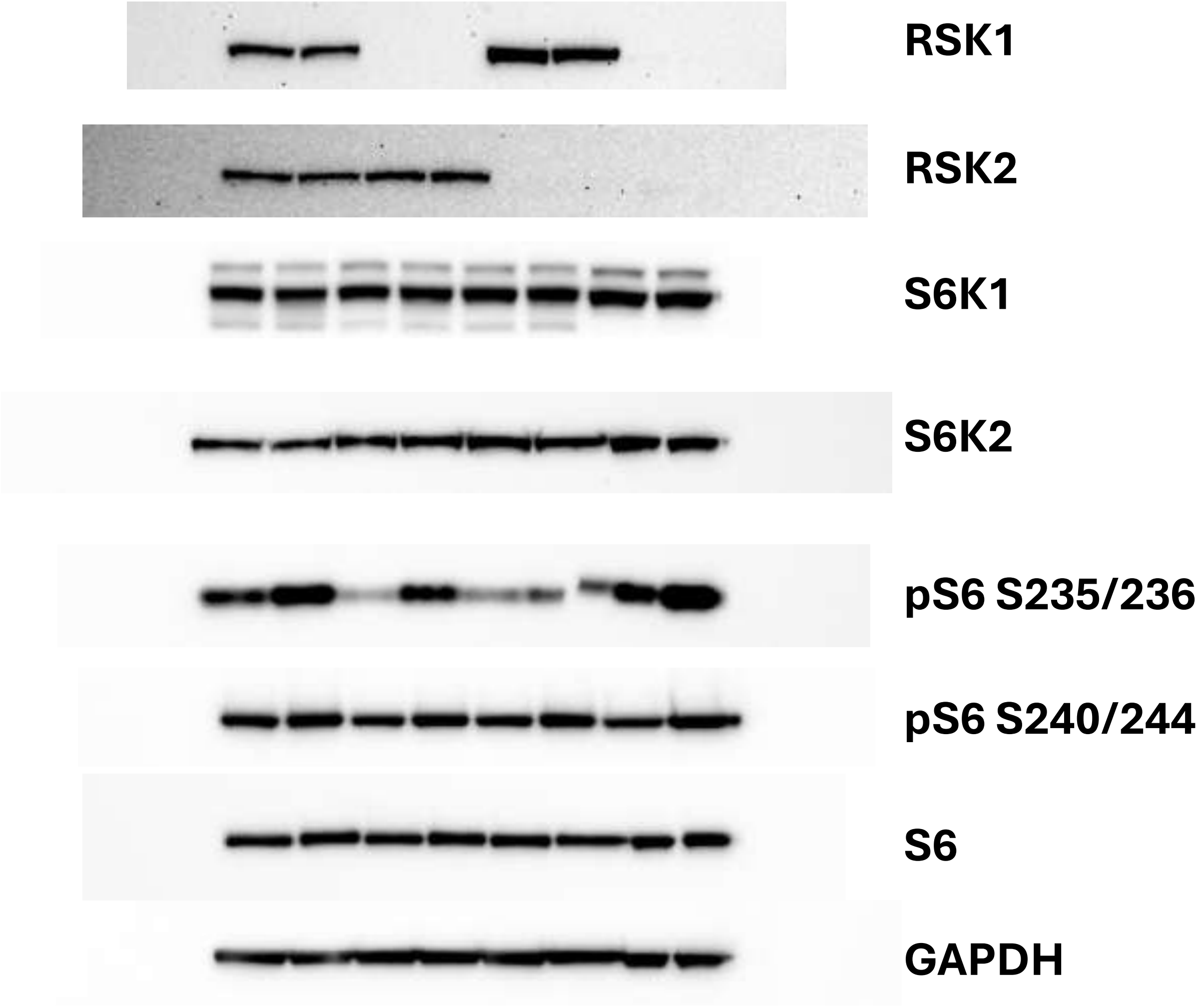

Figure 5B

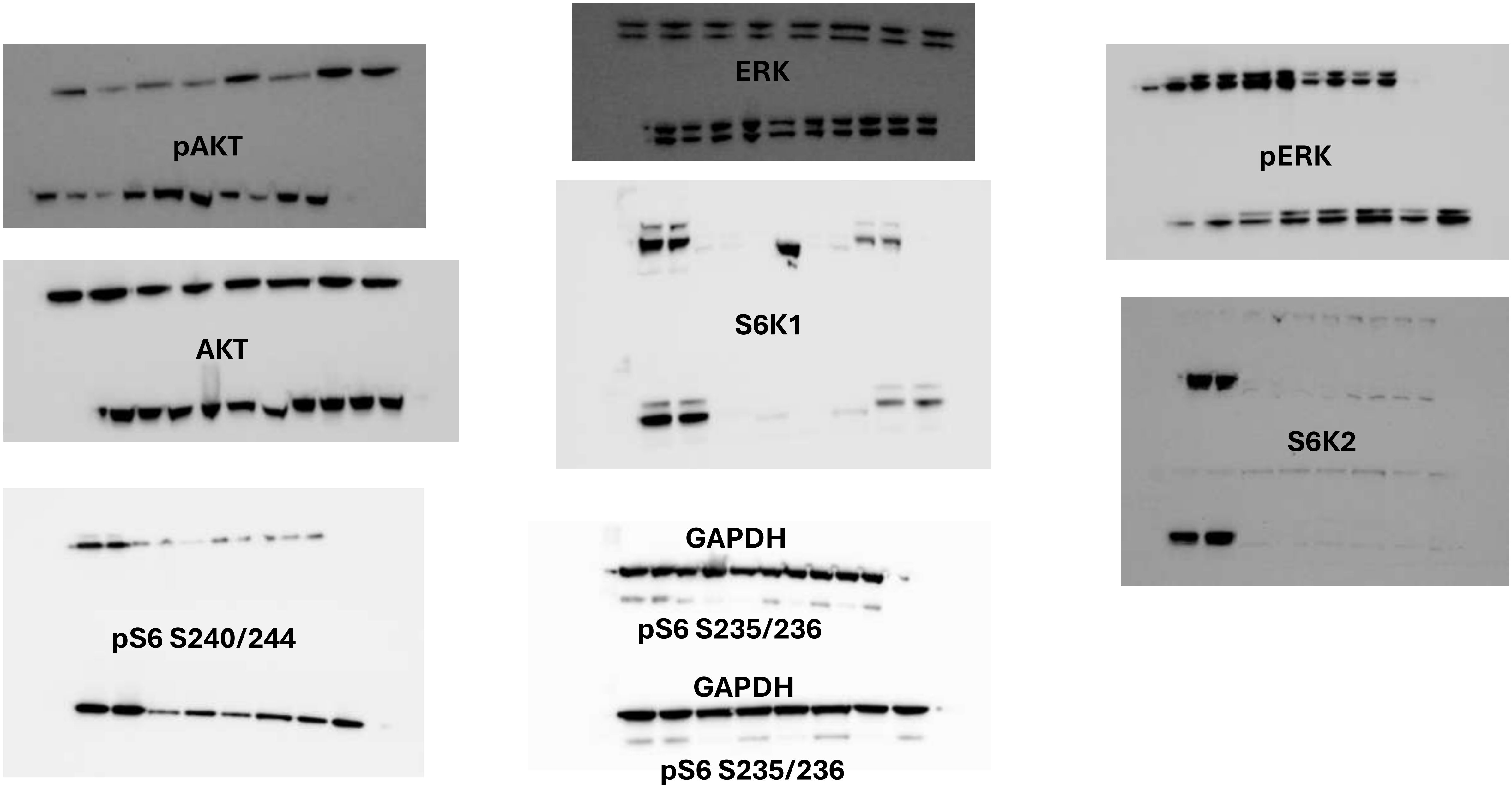
pAKT
AKT
pS6 S240/244
ERK
S6K1
GAPDH
pS6 S235/236
GAPDH
pS6 S235/236
pERK
S6K2

Figure 6B

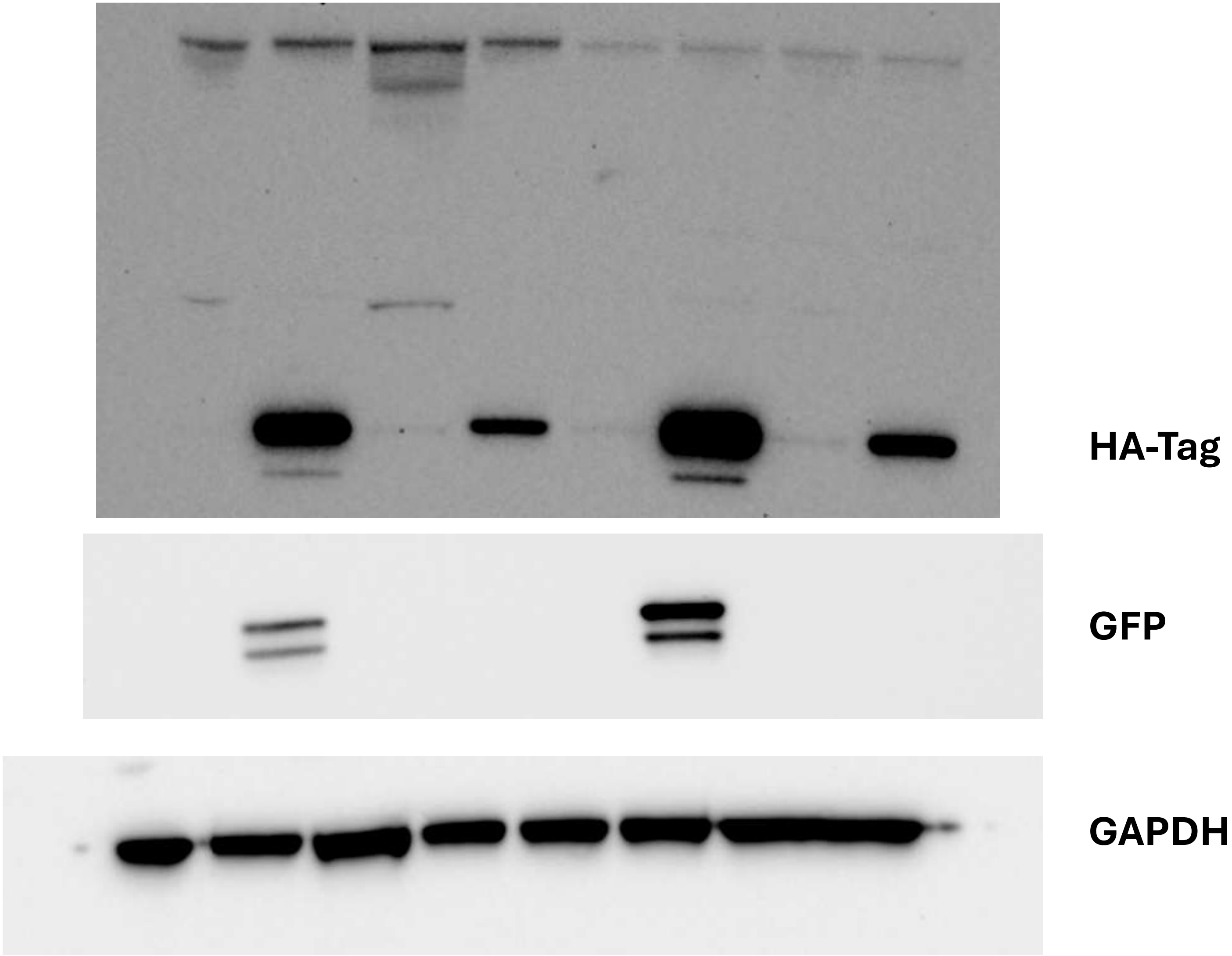

Figure 7D

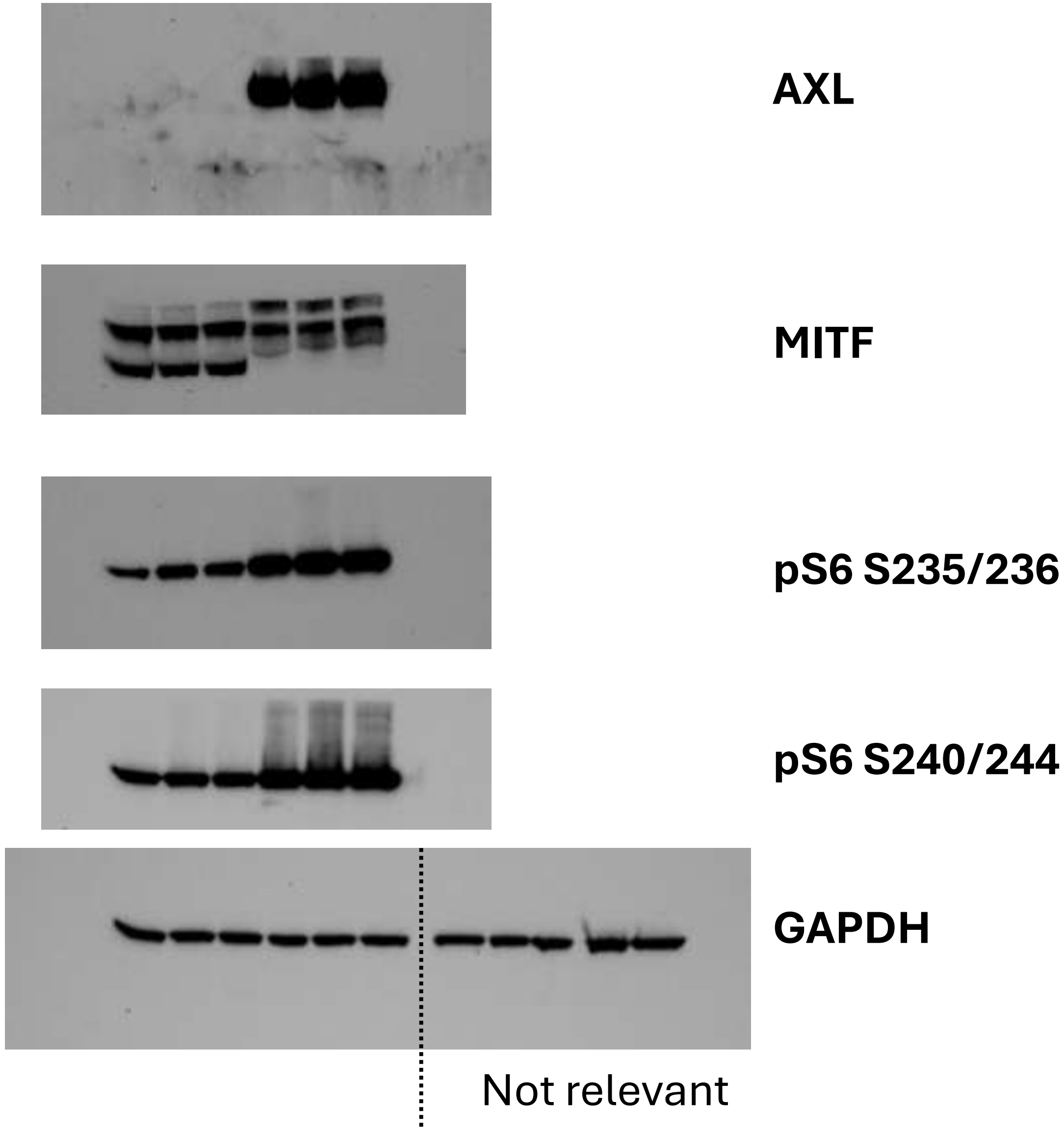

Figure 7E

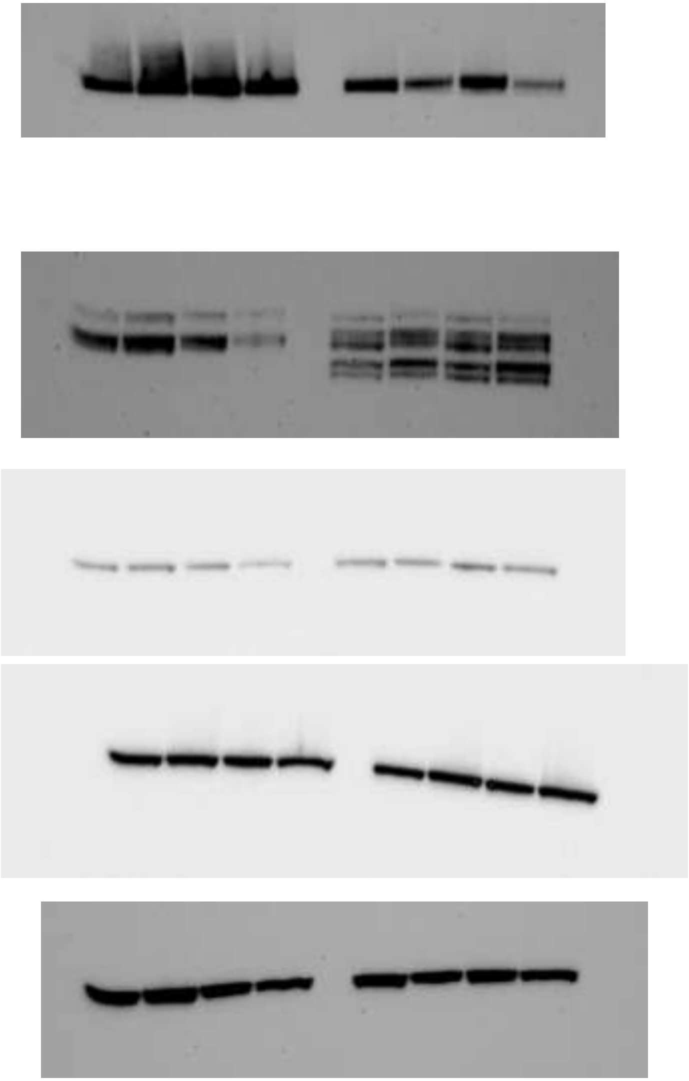

Supplement: Supplementary file 3 — Uncropped western blots [file 41420_2026_3155_MOESM3_ESM.pdf]
