# Supplementary material for: FGFR1 but not S6K1/2 drives intrinsic BRAF inhibitor resistance in melanoma
Source: Cell Death Discov. 2026 May 19;12:294. doi: 10.1038/s41420-026-03155-2 (PMC13350743; doi:10.1038/s41420-026-03155-2)
Supplement: Supplementary file 5 — Supplemental Figure legend [file 41420_2026_3155_MOESM5_ESM.docx]

**Supplemental Figure 1.** BRAFi assay of RPMI7951 and 2686 S6K1/2 single cell cloned DKO cells, crystal violet staining after 4 days of treatment with DMSO or 1uM Vemurafenib.

**Supplemental Figure 2.** Drug dose response matrix for BRAFi (encorafenib) and MEKi (binimetinib) for 3 intrinsically BRAFi-resistant cell lines. Doses are 0nM, 10nM, 30nM, 100nM, 300nM, 1uM. Numbers reflect the normalized OD590 values for crystal violet-stained cells.
